# Biparatopic nanobodies protect mice from lethal challenge with SARS‐CoV‐2 variants of concern

**DOI:** 10.15252/embr.202153865

**Published:** 2021-12-20

**Authors:** Teresa R Wagner, Daniel Schnepf, Julius Beer, Natalia Ruetalo, Karin Klingel, Philipp D Kaiser, Daniel Junker, Martina Sauter, Bjoern Traenkle, Desiree I Frecot, Matthias Becker, Nicole Schneiderhan‐Marra, Annette Ohnemus, Martin Schwemmle, Michael Schindler, Ulrich Rothbauer

**Affiliations:** ^1^ Pharmaceutical Biotechnology Eberhard Karls University Tübingen Germany; ^2^ NMI Natural and Medical Sciences Institute at the University of Tübingen Reutlingen Germany; ^3^ Institute of Virology Medical Center University Freiburg Freiburg Germany; ^4^ Institute for Medical Virology and Epidemiology of Viral Diseases University Hospital Tübingen Tübingen Germany; ^5^ Institute for Pathology and Neuropathology University Hospital Tübingen Tübingen Germany; ^6^ Faculty of Medicine University of Freiburg Freiburg Germany; ^7^ Cluster of Excellence iFIT (EXC2180) “Image‐Guided and Functionally Instructed Tumor Therapies” Eberhard Karls University Tübingen Germany

**Keywords:** human ACE‐2 mouse, neutralizing nanobodies, SARS‐CoV‐2, therapeutics, variants of concern, Immunology, Microbiology, Virology & Host Pathogen Interaction, Molecular Biology of Disease

## Abstract

The ongoing COVID‐19 pandemic and the emergence of new SARS‐CoV‐2 variants of concern (VOCs) requires continued development of effective therapeutics. Recently, we identified high‐affinity neutralizing nanobodies (Nbs) specific for the receptor‐binding domain (RBD) of SARS‐CoV‐2. Taking advantage of detailed epitope mapping, we generate two biparatopic Nbs (bipNbs) targeting a conserved epitope outside and two different epitopes inside the RBD:ACE2 interface. Both bipNbs bind all currently circulating VOCs with high affinities and are capable to neutralize cellular infection with VOC B.1.351 (Beta) and B.1.617.2 (Delta) *in vitro*. To assess if the bipNbs NM1267 and NM1268 confer protection against SARS‐CoV‐2 infection *in vivo*, human ACE2 transgenic mice are treated intranasally before infection with a lethal dose of SARS‐CoV‐2 B.1, B.1.351 (Beta) or B.1.617.2 (Delta). Nb‐treated mice show significantly reduced disease progression and increased survival rates. Histopathological analyses further reveal a drastically reduced viral load and inflammatory response in lungs. These data suggest that both bipNbs are broadly active against a variety of emerging SARS‐CoV‐2 VOCs and represent easily applicable drug candidates.

## Graphical abstract

This study reports on two highly efficient RBD‐specific biparatopic nanobodies that bind and neutralize SARS‐CoV‐2 and its currently circulating variants of concern *in vitro* and *in vivo*, underscoring the potential of nanobodies to prevent or treat SARS‐CoV‐2 infection.

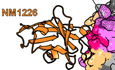

## Introduction

The ongoing SARS‐CoV‐2 pandemic continues to be challenging due to limited access to vaccines in certain countries, vaccine fatigue in others, the lack of effective and easy‐to‐administer antivirals, and the emergence of new variants of concern (VOCs) (Scudellari, 2020). Despite the rapid development of effective vaccines, global immunity or alternatively eradication of SARS‐CoV‐2 is currently out of reach (Kwok *et al*, 2020; Dagan *et al*, 2021). In addition, vaccination does not confer sterile immunity against SARS‐CoV‐2 infection and especially in the elderly, immunocompromised individuals, or individuals with severe preexisting conditions, breakthrough infections can still develop into life‐threatening disease (Beaudoin‐Bussières *et al*, 2020; Long *et al*, 2020; Havlin *et al*, 2021; Kustin *et al*, 2021). In particular, novel variants of concern (VOCs) with increased transmissibility and pathogenicity accompanied by a partial immune escape were reported to cause severe disease progression even in vaccinated individuals (Becker *et al*, 2021; Challen *et al*, 2021; Davies *et al*, 2021a, 2021b; Jewell, 2021; Madhi *et al*, 2021; Volz *et al*, 2021; Zhou *et al*, 2021). Consequently, there is a continuing and urgent need for effective and easily applicable antivirals against emerging VOCs. Neutralizing monoclonal antibodies (Nabs) have been granted emergency use authorization by the U.S. Food and Drug Administration and were shown to efficiently reduce mortality in COVID‐19 patients with increased risk for a severe disease progression (Chen *et al*, 2020; Jiang *et al*, 2020; Weinreich *et al*, 2020). Most of these Nabs target the interaction site between receptor‐binding domain (RBD) of the SARS‐CoV‐2 spike protein and angiotensin‐converting enzyme (ACE) 2 to prevent viral entry into epithelial cells of the respiratory tract (Brouwer *et al*, 2020; Cao *et al*, 2020; Ju *et al*, 2020). However, viral escape from neutralizing antibodies resulted in several mutations affecting the RBD:ACE2 interface, which impairs binding of established Nabs and thus limits current direct‐acting antiviral treatment options (Dejnirattisai *et al*, 2021; preprint: Diamond *et al*, 2021; Wang *et al*, 2021).

In parallel to conventional antibodies, camelid single‐domain antibody fragments, better known as nanobodies (Nbs), have been developed to target the RBD of SARS‐CoV‐2 (Chi *et al*, 2020; Hanke *et al*, 2020; Huo *et al*, 2020; Wrapp *et al*, 2020; Wagner *et al*, 2021). Due to their unique physicochemical properties such as small size, stable folding, and efficient tissue penetration, Nbs are considered to be ideal for therapeutic application. Indeed, some of these Nbs showed strong neutralizing efficacies against SARS‐CoV‐2, especially when used in the multivalent or multiparatopic format (Xiang *et al*, 2020; Huo *et al*, 2021; Koenig *et al*, 2021; Nambulli *et al*, 2021; Schepens *et al*, 2021; Wagner *et al*, 2021).

Recently, we generated a biparatopic (bip) Nb (NM1267) that binds two distinct sites, one epitope inside and one outside of the RBD:ACE2 interface and showed a strong neutralizing capacity (Wagner *et al*, 2021). In this study, we expanded our portfolio for potential therapeutic applications by developing a new bipNb NM1268 that additionally targets a different epitope within the RBD:ACE2 interface. To evaluate their protective efficacy *in vivo*, transgenic mice expressing human ACE2 (K18‐hACE2 mice) (McCray *et al*, 2007; Winkler *et al*, 2020) were challenged with a lethal dose of the early circulating SARS‐CoV‐2 B.1, VOC B.1.351 (Beta) or VOC B.1.617.2 (Delta). Consistent with its neutralizing activity *in vitro*, NM1267 efficiently protected mice from weight loss and profound lung tissue damage after infection with SARS‐CoV‐2 B.1 or VOC B.1.351 (Beta), whereas NM1268 was slightly more protective against the VOC B.1.617.2 (Delta). This demonstrates how well‐characterized Nbs targeting different functional epitopes can be combined as bipNbs to serve as promising drug candidates.

## Results

**Figure EV1:**
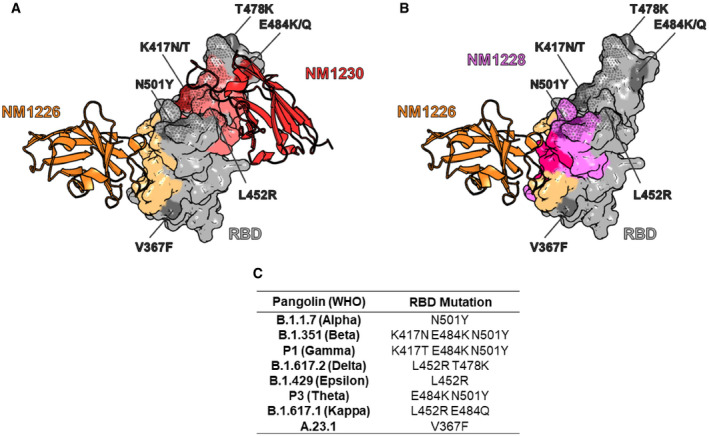
Influence of RBD mutations on bipNb binding NM1267‐binding epitope on RBD derived from crystal structure of single RBD:Nb complexes NM1226 (orange, PDB 7NKT) and NM1230 (red, PDB 7B27) (Wagner *et al*, 2021). NM1267‐forming single Nbs are shown as cartoon with their corresponding binding epitopes on the RBD surface in light orange and light red, respectively. In addition, the ACE2 interaction site on RBD is illustrated as dotted surface. Mutations on RBD of identified SARS‐CoV‐2 variants, including B.1.1.7 (Alpha), B.1.351 (Beta), P1 (Gamma), B.1.617.2 (Delta), B.1.429 (Epsilon), P3 (Theta), B.1.617.1 (Kappa), and A.23.1 are highlighted in dark gray or dark red and labeled, respectively.NM1268‐binding epitope on RBD derived from crystal structure of the RBD:Nb complex for NM1226 (orange, PDB 7NKT) and HDX‐MS analysis for NM1228 (purple) (Wagner *et al*, 2021). NM1268‐forming single Nb NM1226 is shown as cartoon with its corresponding binding epitope on the RBD surface in light orange. Binding epitope of NM1228 is illustrated in purple, while shared binding sites of NM1226 and NM1228 are illustrated in pink. In addition, the ACE2 interaction site on RBD is illustrated as a dotted surface. Mutations on RBD of identified VOCs, including B.1.1.7 (Alpha), B.1.351 (Beta), P1 (Gamma), B.1.617.2 (Delta), B.1.429 (Epsilon), P3 (Theta), B.1.617.1 (Kappa), and A.23.1 are highlighted in dark gray or dark purple and labeled, respectively.Table summarizing mutations on RBD of different SARS‐CoV‐2 VOCs.

**Figure 1:**
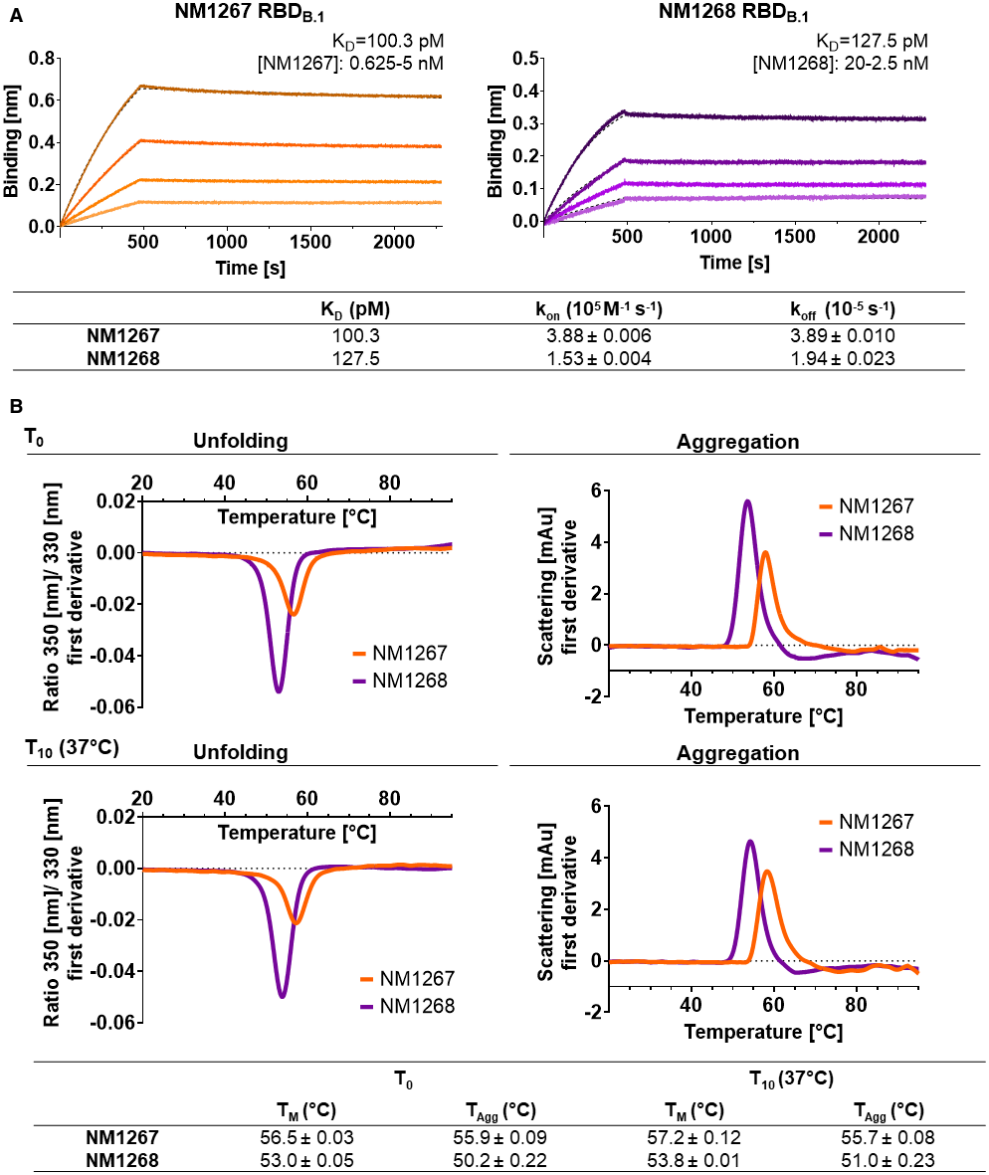
Affinity and stability of different biparatopic nanobodies Affinity measurements by biolayer interferometry (BLI) of bipNbs NM1267 and NM1268. NM1267 and NM1268 were applied with concentrations ranging from 5 to 0.625 nM and from 20 to 2.5 nM, respectively (illustrated with gradually lighter shades) on immobilized RBD derived from SARS‐CoV‐2 B.1 (RBD_B.1_). Global 1:1 fits are illustrated as dashed lines and binding affinity (*K*_D_), association (*k*_on_), and dissociation constant (*k*_off_) determined for the individual bipNbs are summarized.Stability analysis of bipNbs NM1267 and NM1268 was performed at time points *T*_0_ and *T*_10_ after storage at 37°C for 10 days to induce accelerated aging. Protein unfolding was determined by fluorescence emission wavelength shifts illustrated as fluorescence ratios (350 nm/ 330 nm) as first derivative. Protein aggregation status was measured by light intensity loss due to scattering illustrated as first derivative. Melting (*T*_m_) and aggregation (*T*_Agg_) temperature are summarized as table for both time points.

**Figure EV2:**
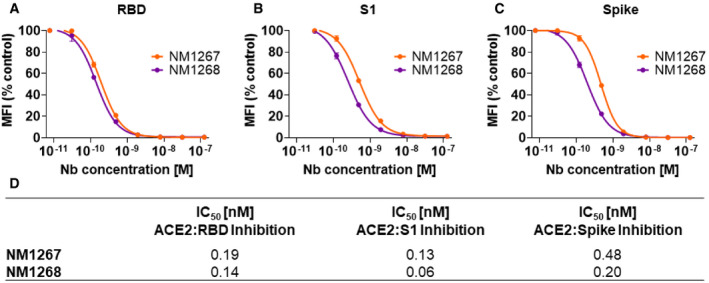
Biparatopic NM1267 and NM1268 compete with ACE2 A–CResults from multiplex ACE2 competition assay are shown for the three spike‐derived antigens: RBD (A), S1‐domain (S1) (B), and homotrimeric spike (Spike) (C). Color‐coded beads coated with the respective antigens were co‐incubated with biotinylated ACE2 and dilution series of NM1267 and NM1268 (8 pM to 126 nM) followed by measuring residual binding of ACE2. MFI signals were normalized to the maximum detectable signal per antigen given by the ACE2‐only control. IC_50_ values were calculated from a four‐parametric sigmoidal model. Data are presented as mean ± SD of three technical replicates.DTable summarizing IC_50_ values of the multiplex ACE2 competition assay obtained for NM1267 and NM1268.

Following our recently reported approach in which we combined two SARS‐CoV‐2 RBD‐binding Nbs to generate the strongly neutralizing biparatopic Nb (bipNb) NM1267 (Wagner *et al*, 2021), we designed an additional bipNb by genetically coupling the neutralizing Nbs NM1228 and NM1226 via a flexible Gly‐Ser ((G_4_S)_4_) linker (Appendix Table S1). While the bipNb NM1267 combines the two Nbs NM1230 and NM1226, which have been shown to target two distinct epitopes, one inside and one outside the RBD:ACE2 interface, the new bipNb NM1268 includes, in addition to NM1226, Nb NM1228, which binds a different epitope inside the RBD:ACE2 interface (Fig EV1A and B). Like NM1267 (Wagner *et al*, 2021), NM1268 was produced with high yield and good purity in mammalian cells and showed picomolar affinities to RBD derived from SARS‐CoV‐2 B.1 (RBD_B.1_) as measured by biolayer interferometry (BLI; Fig 1A). The results of a multiplex ACE2 competition assay (Wagner *et al*, 2021) further revealed that both bipNbs block the interaction of SARS‐CoV‐2 RBD, S1, or homotrimeric Spike and human ACE2 in a low picomolar range (Fig EV2A–D). For further analysis, we next assessed their biophysical properties by measuring thermal unfolding and aggregation with nano differential scanning fluorimetry (nanoDSF; Fig 1B). While both bipNbs showed a slight increase in light scattering, indicating a higher aggregation tendency at higher, non‐physiological temperatures, reanalysis after accelerated aging at 37°C for 10 days revealed no considerable differences compared to baseline (Fig 1B). From these data, we concluded that both bipNbs are highly stable and applicable for further *in vivo* analysis.

**Figure 2:**
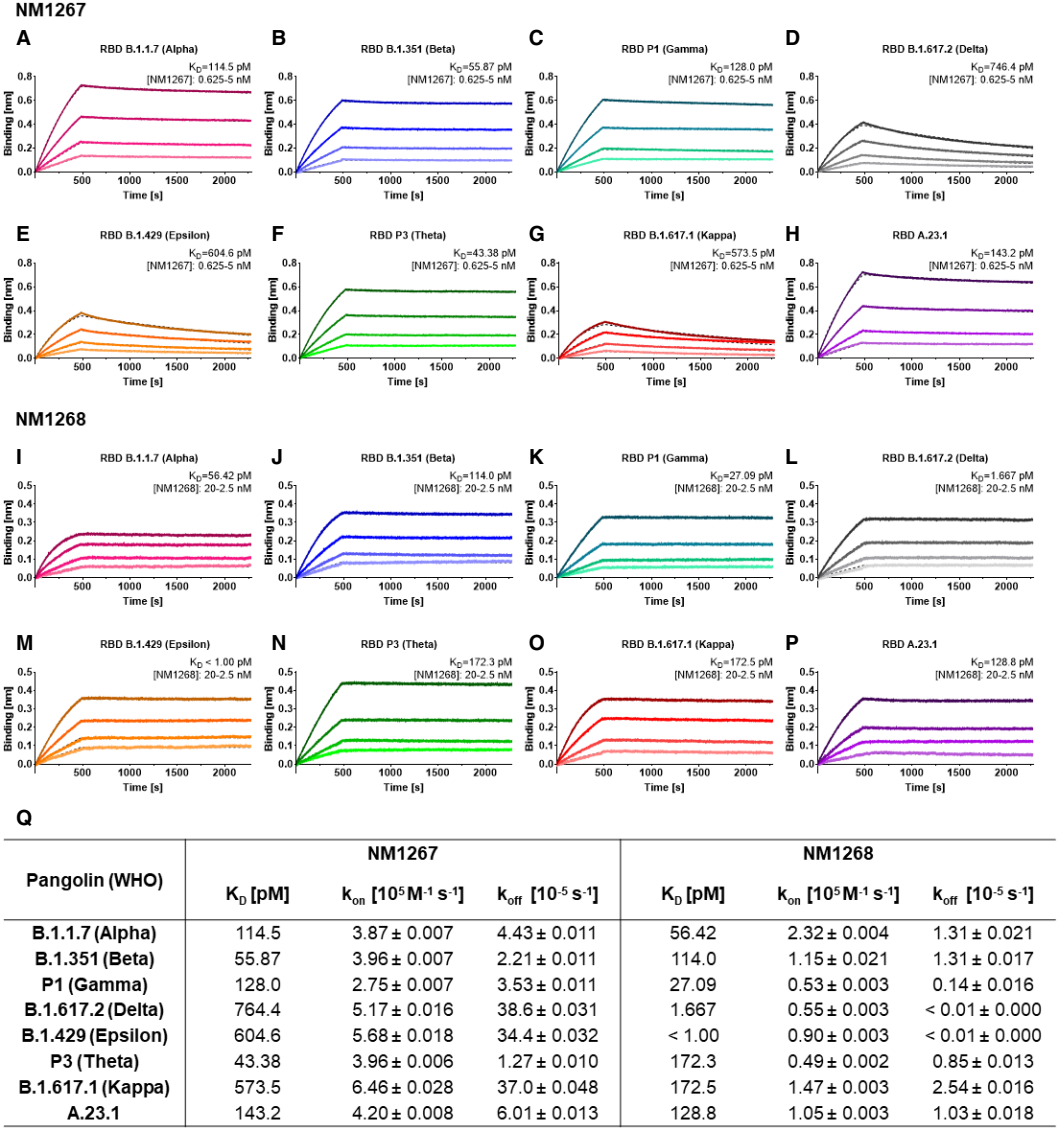
Biparatopic Nanobodies target several recently identified RBD variants with picomolar affinity A–PAffinity measurements by BLI of bipNb NM1267 and NM1268 on recently identified RBD variants B.1.1.7 (Alpha) (A, I), B.1.351 (Beta) (B, J), P1 (Gamma) (C, K), B.1.617.2 (Delta) (D, L), B.1.429 (Epsilon) (E, M), P3 (Theta) (F, N), B.1.617.1 (Kappa) (G, O), and A.23.1 (H, P). NM1267 and NM1268 were applied with concentrations ranging from 5 to 0.625 nM and from 20 to 2.5 nM, respectively (illustrated with gradually lighter shades), on immobilized RBD variants. Global 1:1 fits are illustrated as dashed lines.QTabular summary of binding affinity (*K*_D_), association (*k*_on_), and dissociation constant (*k*_off_) determined for the individual RBD variants.

With the precise epitopes known, we considered that both bipNbs could also be effective against lately described VOCs (Fig EV1A–C). Therefore, we analyzed binding affinities of NM1267 and NM1268 toward RBDs of emerging SARS‐CoV‐2 variants using BLI (Fig 2A–Q). Compared to RBD of B.1, NM1267 showed similar or even increased affinity to RBDs from B.1.1.7 (Alpha; Fig 2A), B.1.351 (Beta; Fig 2B), P1 (Gamma; Fig 2C), P3 (Theta; Fig 2F) and A.23.1 (Fig 2H). A slight decrease in affinity was observed for RBDs from B.1.617.2 (Delta; Fig 2D), B.1.429 (Epsilon; Fig 2E) and B.1.617.1 (Kappa; Fig 2G), all of which have the exchange of Leu for Arg at position 452 (L452R). In contrast, NM1268 displayed robust binding affinities to all measured VOCs (Fig 2I–Q), with remarkable ~ 100‐fold increased binding affinities compared to other VOCs determined for B.1.617.2 (Delta; Fig 2L) and B.1.429 (Epsilon; Fig 2M). In summary, the measured affinities confirmed the high potential of NM1267 and NM1268 to efficiently bind SARS‐CoV‐2 variants with multiple mutations at different positions of the RBD (Figs 2A–Q and EV1A–C).

**Figure 3:**
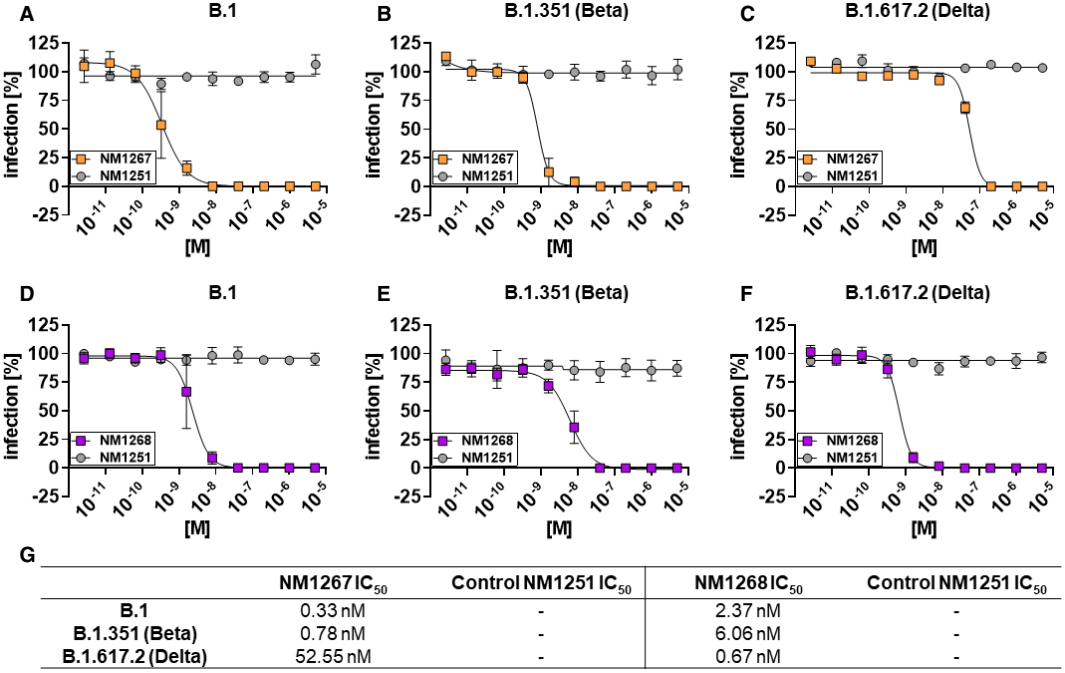
Biparatopic Nanobodies neutralize B.1, B.1.351, and B.1.617.2 SARS‐CoV‐2 infection in Caco‐2 cells A–FNeutralization potency of NM1267 and NM1268 were analyzed in Caco‐2 cells using the SARS‐CoV‐2 B.1 (A, D) SARS‐CoV‐2 B.1.351 (Beta) (B, E), and SARS‐CoV‐2 B.1.617.2 (Delta) (C, F). Infection normalized to virus‐only infection control is illustrated as percent of infection (infection [%]). Data are presented as mean ± SEM of three (*n* = 3) biological replicates.GTabular summary of IC_50_ values, calculated from a four‐parametric sigmoidal model.

The VOCs can evade the immune response after vaccination or treatment with established Nabs (Becker *et al*, 2021; Madhi *et al*, 2021; Mlcochova *et al*, 2021; Wang *et al*, 2021), due to escape mutations within the RBD (Li *et al*, 2021; Zhou *et al*, 2021). To investigate the neutralization capacity of NM1267 and NM1268 against the two escape variants B.1.351 (Beta) and B.1.617.2 (Delta) *in vitro*, we performed virus neutralization assays (VNTs) using a non‐specific bivalent Nb (bivNb) NM1251 as negative control. For NM1267, we observed a strong neutralization of SARS‐CoV‐2 B.1 and B.1.351 (Beta), with IC_50_ values of 0.33 and 0.78 nM, respectively. Still efficient, albeit lower neutralization was determined for the B.1.617.2 (Delta) variant with an IC_50_ value of 52.55 nM, a finding that was consistent with the decreased affinity measured for all VOCs harboring the L452R mutation (Fig 3A–C and G). In contrast, a strong neutralization potency of NM1268 was determined for all variants with IC_50_ values of 2.37 nM for SARS‐CoV‐2 B.1, 6.06 nM for B.1.351 (Beta), and 0.67 nM for B.1.617.2 (Delta; Fig 3D–G), in line with the measured affinities.

**Figure 4:**
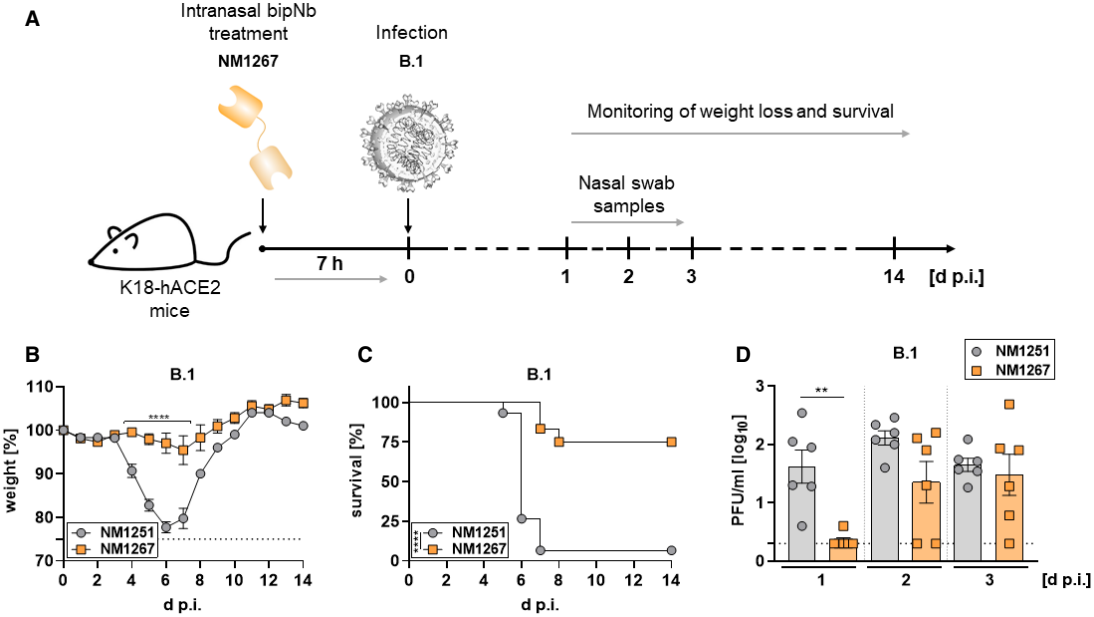
Intranasal application of NM1267 protects K18‐hACE2 mice against SARS‐CoV‐2 B.1‐induced disease and reduces mortality and virus shedding ASchematic illustration of treatment scheme.B, CHemizygous K18‐hACE2 mice were treated intranasally with 20 µg of NM1251 (*n* = 15) or NM1267 (*n* = 12) 7 h prior to infection with 3 × 10^3^ PFU SARS‐CoV‐2 B.1. Weight loss (B) and survival (C) were monitored for 14 days.DNasal swabs were collected from six mice per group (*n* = 6) at the indicated time points and viral load was determined by plaque‐assay. Data information: Dashed line indicates humane end point in (B) and symbols represent mean ± SEM in (B) or individual animals in (D). Bars in (D) represent mean ± SEM. ****P < 0.0001, by two‐way ANOVA with Sidak's multiple comparison test in (B), ****P < 0.0001, by log‐rank test in (C), and **P < 0.01, by unpaired *t*‐test in (D).

**Figure 5:**
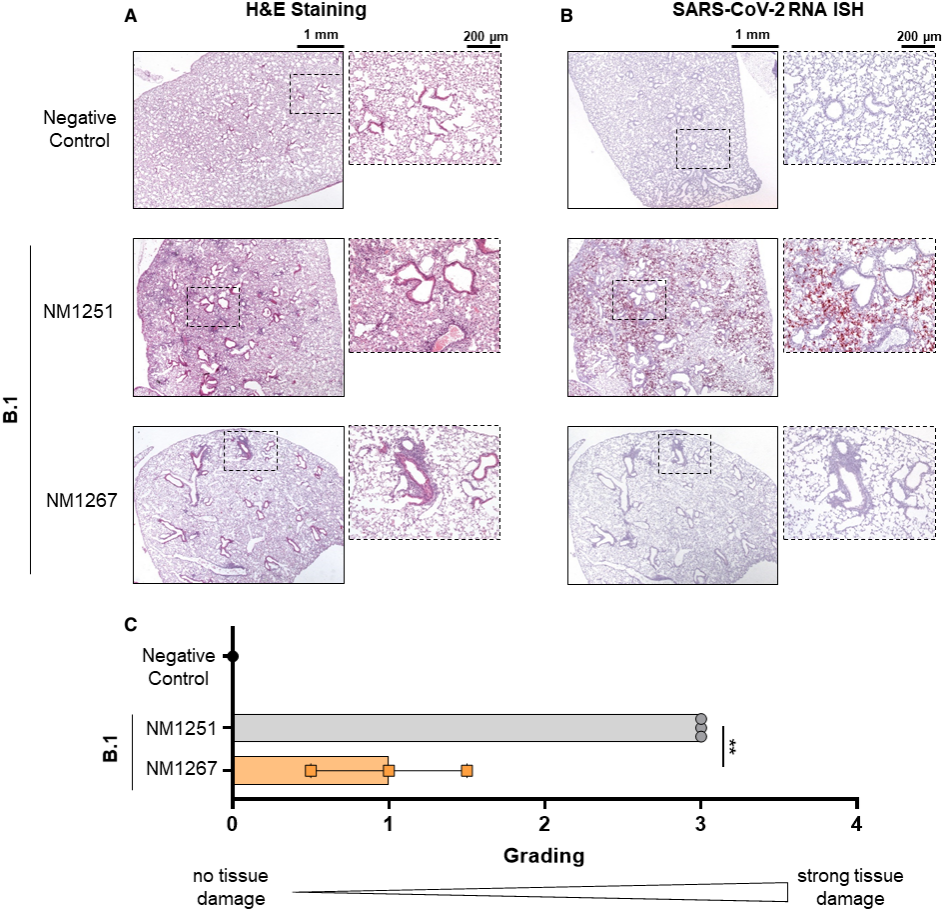
Microscopic analysis of lung tissue from SARS‐CoV‐2‐infected K18‐hACE2 mice Histopathological analysis of mice, which were intranasally treated with bipNb NM1267 or control Nb NM1251 and subsequently infected with 3 × 10^3^ PFU SARS‐CoV‐2 B.1.
A, BSerial tissue sections revealed severe inflammation (H&E) and numerous widespread SARS‐CoV‐2 RNA‐positive alveolar epithelia cells and macrophages (*in situ* hybridization [ISH]) in lungs of infected, control‐treated mice. In infected and NM1267‐treated animals no inflammation or only small focal areas with inflammation and a few SARS‐CoV‐2 RNA‐positive cells were observed. Scale bars represent 1 mm and 200 µm, respectively.CQuantitation of lung damage was done in *n* = 3 animals per group and grading score of individual animals is presented as mean ± SD with **P < 0.01, by unpaired *t*‐test.

Next, we evaluated the efficacy of bipNbs as potential therapeutics to combat infections with SARS‐CoV‐2 *in vivo*. Therefore, we used K18‐hACE2 transgenic mice expressing human ACE2 which are highly permissive for infection with clinical SARS‐CoV‐2 isolates (Winkler *et al*, 2020). Considering a broad applicability for which noninvasive routes of administration are preferred, we chose to deliver the bipNbs intranasally. In an initial experimental setting, mice were treated prophylactically with 20 µg NM1267 or the non‐specific control (NM1251) followed by SARS‐CoV‐2 B.1 infection 7 h later (Fig 4A). Weight loss and survival of infected mice were monitored for 14 days post‐infection (d p.i.). All infected animals treated with the negative control NM1251 became severely sick with obvious clinical signs of disease, lost substantial amounts of body weight, and 14 out of 15 animals had to be euthanized (Fig 4B and C). In contrast, administration of NM1267 significantly reduced signs of disease, weight loss, and 9 out of 12 animals survived the infection. Additionally, virus shedding by NM1267‐treated mice, determined by viral load on nasal swabs, was significantly reduced in comparison with control animals on day 1 after infection (Fig 4D). We further performed histopathological analyses of lungs from SARS‐CoV‐2 B.1‐infected mice treated with either NM1251 or NM1267. Hematoxylin and eosin (H&E) staining was performed to evaluate the degree of tissue damage upon infection, and *in situ* hybridization (ISH) was used to visualize the extent and localization of viral RNA. Applying a grading system from 0 (no tissue damage) to 4 (strong tissue damage), it became evident that all SARS‐CoV‐2‐infected mice under control treatment (NM1251) exhibited a pronounced inflammation and loss of functional lung epithelia (Fig 5A–C). In contrast, prophylactic treatment with NM1267 efficiently reduced virus‐ and inflammation‐induced tissue damage within the lungs (scoring 0.5–1.5) of SARS‐CoV‐2 B.1‐infected mice (Fig 5A–C). In line with these findings, distinctly lower levels of SARS‐CoV‐2 RNA were found in samples taken from NM1267‐treated mice, restricted to minimal areas of the lung at sub‐pleural position and some fat cells. Analysis of lung sections of control‐treated mice showed widespread presence of virus RNA‐positive epithelial cells (Fig 5B).

**Figure EV3:**
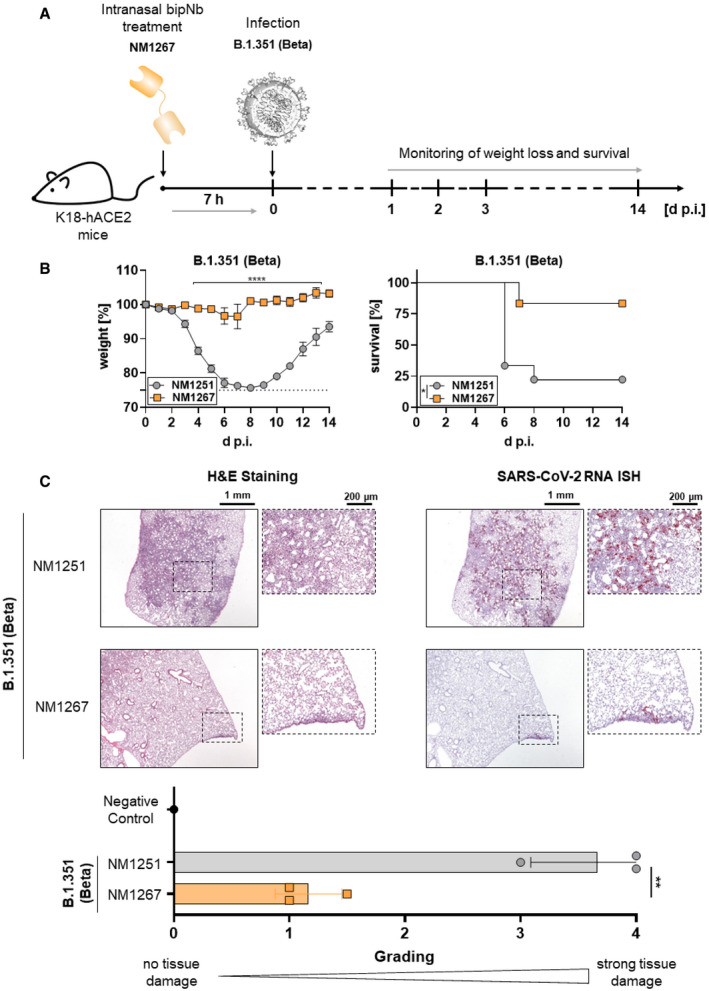
Intranasal application of NM1267 protects K18‐hACE2 mice against B.1.351 (Beta)‐induced disease, reduces mortality, and lung tissue damage Schematic illustration of treatment scheme.Hemizygous K18‐hACE2 mice were treated intranasally with 20 µg of NM1251 (*n* = 9) or NM1267 (*n* = 6) 7 h prior to infection with 3 × 10^3^ PFU SARS‐CoV‐2 B.1.351 (Beta). Weight loss (left) and survival (right) were monitored for 14 days. Dashed line indicates humane end point and symbols for weight loss (left) represent mean ± SEM. ****P < 0.0001, by two‐way ANOVA with Sidak's multiple comparison test (left) and *P < 0.05, by log‐rank test for survival (right).Serial tissue sections revealed severe inflammation (H&E) and numerous widespread SARS‐CoV‐2 RNA‐positive alveolar epithelia cells and macrophages (*in situ* hybridization [ISH]) in lungs of infected, control‐treated mice. In infected and NM1267 bipNb‐treated animals no inflammation or only small focal areas with inflammation and a few SARS‐CoV‐2 RNA‐positive cells were observed. A negative control is displayed in Fig 5A and B. Scale bars represent 1 mm and 200 µm, respectively. Quantitation of lung damage was done in *n* = 3 animals per group and grading score of individual animals is presented as mean ± SD with **P < 0.01, by unpaired *t*‐test.

Prophylactic treatment with NM1267 was equally potent in blocking disease progression if VOC B.1.351 (Beta) was used to infect mice. Only one NM1267‐treated mouse infected with B.1.351 (Beta) lost substantial amounts of weight and had to be euthanized, whereas five out of six animals did not show any signs of disease and survived the infection (Fig EV3A and B). Similarly, histopathological examinations demonstrated a significant reduction of virus replication and tissue damage by NM1267 treatment (Fig EV3C).

**Figure 6:**
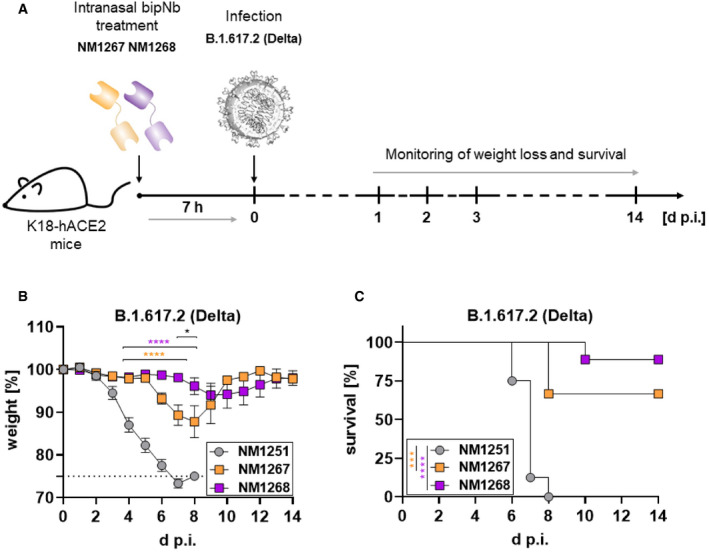
Intranasal application of NM1267 and NM1268 protects K18‐hACE2 mice against VOC B.1.617.2‐induced disease and reduces mortality ASchematic illustration of treatment scheme.B, CHemizygous K18‐hACE2 mice were treated intranasally with 20 µg of NM1251 (*n* = 8), NM1267 (*n* = 9) or NM1268 (*n* = 9) 7 h prior to infection with 3 × 10^3^ PFU SARS‐CoV‐2 B.1.617.2 (Delta). Weight loss (B) and survival (C) were monitored for 14 days. Data information: Dashed line indicates humane end point and symbols represent mean ± SEM in (B). ****P < 0.0001 in orange between NM1251 and NM1267 and in purple between NM1251 and NM1268, *P < 0.1 in black between NM1267 and NM1268, by two‐way ANOVA with Sidak's multiple comparison test in (B). ***P < 0.001 in orange between NM1251 and NM1267 and ****P < 0.0001 in purple between NM1251 and NM1268, by log‐rank test in (C).

After demonstrating the general applicability of bipNbs for prophylactic treatment, we performed a comparative study of the two bipNbs NM1267 and NM1268 with respect to their potential to block disease progression upon infection with the currently predominant VOC B.1.617.2 (Delta). Hence, mice were treated with 20 µg of either NM1267 or NM1268 followed by infection with the B.1.617.2 (Delta; Fig 6A). Similar to SARS‐CoV‐2 B.1 and B.1.351 (Beta), all NM1251‐treated animals infected with B.1.617.2 became severely ill and reached humane end points between day 6 and day 8 post‐infection. In contrast, the majority of animals treated with bipNbs NM1267 and NM1268 survived the infection. In direct comparison and consistent with the VNT data, NM1268 showed a slightly stronger protective effect against B.1.617.2 (Delta) compared with NM1267, as only one out of nine animals treated with NM1268 had to be euthanized in contrast to three out of nine mice in the NM1267‐treated group (Fig 6B and C).

In conclusion, the *in vivo* data demonstrate that prophylactic intranasal administration of bipNbs NM1267 and NM1268 can efficiently prevent disease progression and mortality caused by SARS‐CoV‐2 B.1, B.1.351 (Beta), and B.1.617.2 (Delta). This highlights the potential of both bipNbs as broadly applicable drug candidates to prevent or treat SARS‐CoV‐2 infections in unvaccinated or immunocompromised individuals at high risk of developing severe disease.

## Discussion

In this study, we investigated the potential of two biparatopic Nbs, NM1267 and NM1268, targeting different epitopes within the RBD, to prevent SARS‐CoV‐2‐induced disease and mortality. Biochemical analyses of NM1267 and NM1268 demonstrated vigorous ACE2 displacement, high thermal stabilities, and strong binding to all SARS‐CoV‐2 RBD variants tested. Importantly, we could show profound neutralizing capacity of both bipNbs against SARS‐CoV‐2 B.1 and two immune‐evading VOCs, B.1.351 (Beta) and B.1.617.2 (Delta).

Prophylactic administration of both NM1267 or NM1268 *in vivo*, strongly diminished disease progression and mortality in SARS‐CoV‐2 B.1, B.1.351 (Beta)‐ or B.1.617.2 (Delta)‐infected transgenic mice. Overall, these data underscore the potential of both bipNbs to prevent or treat infections with VOCs for which currently available vaccines and therapeutic approaches are suspected to have reduced efficacy (Becker *et al*, 2021; Kustin *et al*, 2021; Madhi *et al*, 2021; Planas *et al*, 2021; Zhou *et al*, 2021). Histopathological analyses and *in situ* hybridization detecting viral RNA in lung tissue samples further revealed significantly reduced tissue damage and virus replication, suggesting that bipNb treatment may also reduce long‐term effects of SARS‐CoV‐2 infections (Han *et al*, 2021; Yong, 2021).

Notably, most strategies for engineering neutralizing Nbs currently rely on increasing avidity by generating multivalent constructs binding the same epitope (Nambulli *et al*, 2021; Schepens *et al*, 2021; Wu *et al*, 2021), whereas only very few reports are available in which two Nbs targeting different epitopes on RBD were combined to prevent virus escape (preprint: Hanke *et al*, 2021; Koenig *et al*, 2021). Administration of NM1267 and NM1268 showed strong short‐term efficacy *in vivo*. Additional modifications like fusion to an Fc‐moiety, addition of an albumin‐binding motif or direct linkage to carrier proteins like albumin may be beneficial to improve duration of effectiveness (preprint: Hanke *et al*, 2021; Huo *et al*, 2021; Nambulli *et al*, 2021; Schepens *et al*, 2021; Wu *et al*, 2021). Moreover, the extraordinary susceptibility of transgenic hACE2 mice to SARS‐CoV‐2‐induced disease due to the artificial overexpression of hACE2 in a variety of tissues and organs, may even result in an underestimated therapeutic potential of both bipNbs. To address this issue and to investigate the potential of NM1267 and NM1268 to prevent virus transmission, further studies in more physiological models, such as Syrian hamsters or non‐human primates, will be required (Haga *et al*, 2021; Nambulli *et al*, 2021; Schepens *et al*, 2021). Additionally, in terms of clinical translation the immunogenicity profile of both bipNbs needs to be evaluated. Notably, it has recently been shown that clinically applied Nbs are only weakly immunogenic (Ackaert *et al*, 2021). Furthermore, strategies for humanization at selected positions are available (Muyldermans *et al*, 2009; Vincke *et al*, 2009).

In summary, this study demonstrates how rational design based on detailed knowledge of binding properties, recognized epitopes, and *in vitro* determination of neutralization capacities can be combined to develop drug candidates that blocks SARS‐CoV‐2‐induced disease and mortality *in vivo*. Given the restricted access to vaccines in various countries, vaccination fatigue, and the frequent emergence of new variants of concern, we believe that the development of such easily applicable therapeutic approaches to protect and treat predisposed individuals are highly promising strategies and urgently warranted.

## Materials and Methods

### Expression constructs

To generate described expression constructs, all used primer sequences are listed in Appendix Table S2. Nb NM1267 was generated as described previously (Wagner *et al*, 2021). BipNb NM1268 composed of Nb NM1228 and Nb NM1226 (Wagner *et al*, 2021) was similarly generated using primers NM1228Nfor, NM1228Nrev and NM1226Cfor, NM1226Crev. DNA coding for bipNbs were cloned into pCDNA3.4 expression vector seamlessly downstream of comprising N‐terminal signal peptide (MGWTLVFLFLLSVTAGVHS) for secretory pathway using type IIS restriction enzyme Esp3I and EcoRI site. Coding sequence of bivNb NM1251 (Traenkle *et al*, 2020) was produced by gene synthesis (Thermo Fisher Scientific) and similarly cloned into pCDNA3.4 expression vector. Receptor‐binding domain (RBD) variants of SARS‐CoV‐2 were generated as earlier published (Wagner *et al*, 2021). The expression plasmid pCAGGS encoding the RBD of SARS‐CoV‐2 spike protein (amino acids 319–541) was kindly provided by F. Krammer. RBDs of SARS‐CoV‐2 variants of concern (VOCs) B.1.1.7 (Alpha), B.1.351 (Beta), P1 (Gamma), B.1.617.2 (Delta), B.1.429 (Epsilon), P3 (Theta), B.1.617.1 (Kappa), and A.23.1 were generated by PCR amplification of fragments from B.1 or cognate DNA template and subsequent fusion PCR by overlap extension to introduce described mutations. Based on RBD_B.1_ sequence primer pairs RBDfor N501Yrev and N501Yfor RBDrev were used for the amplification of B.1.1.7 (Alpha) sequence; primer pairs RBDfor L452Rrev and L452Rfor RBDrev for B.1.429 (Epsilon); RBDfor V367Frev and V367F for RBDrev for A.23.1. B.1.617.2 (Delta) was generated based on B.1.429 (Epsilon) using primer pairs RBDfor T478Krev and T478Kfor RBDrev. Based on B.1.1.7 (Alpha) sequence P3 (Theta) was generated using primer pairs RBDfor E484Krev and E484Kfor RBDrev. B.1.617.1 (Kappa) was generated using primer pairs RBDfor E484Krev and E484Kfor RBDrev as well as RBDfor L452Rrev and L452Rfor RBDrev. B.1.351 (Beta) and P1 (Gamma) were generated based on P3 (Theta) sequence using primer pairs RBDfor K417Nrev and K417Nfor RBDrev; and RBDfor K417Trev and K417Tfor RBDrev, respectively. Amplicons were inserted using XbaI and NotI site into the pCDNA3.4 expression vector. The integrity of all expression constructs was confirmed by standard sequencing analysis.

### Protein expression and purification

Confirmed constructs were expressed in Expi293 or ExpiCHO cells and transfection was performed as per the manufacturer's instructions (Thermo Fisher Scientific). Cell suspensions were then cultivated for 2–5 days (37°C, 125 rpm, 8% (v/v) CO_2_) and then centrifuged (4°C, 23,900 *g*, 20 min) to clarify the supernatant. Supernatants were then filtered with a 0.22‐µm membrane (Millipore) and supplemented with His‐A buffer stock solution (20 mM Na_2_HPO_4_, 30 mM NaCl, 20 mM imidazole, pH 7.4). The solution was then applied to a HisTrap FF crude column on an Aekta pure system (GE Healthcare), extensively washed with His‐A buffer, and eluted with an imidazole gradient (50–400 mM). Buffer exchange to PBS and concentration of eluted proteins were carried out using Amicon 10 K centrifugal filter units (Millipore). Protein quality was analyzed by standard SDS–PAGE and via the NanoDrop protein concentration was determined.

### Affinity measurements

Binding affinity of bipNbs toward variants of RBD was determined via biolayer interferometry (BLI) using the Octet RED96e system according to standard protocol. Therefore, RBD variants were biotinylated and immobilized on streptavidin biosensors (SA, Sartorius). Dilution series ranging from 20 to 0.625 nM of bipNbs were applied and one reference was included per run. For affinity determination, the 1:1 global fit of the Data Analysis HT 12.0 software was used.

### Bead‐based multiplex ACE2 competition assay

To analyze binding competition of human ACE2 versus generated bipNbs the bead‐based multiplex ACE2 competition assay was performed as previously described (Wagner *et al*, 2021).

### Stability analysis

Stability analysis was performed by the Prometheus NT.48 (Nanotemper). Therefore, freshly thawed bipNbs were diluted to 0.25 mg/ml and measurements were carried out at time point T_0_ and after incubation for 10 days at 37°C (T_10_) using high‐sensitivity capillaries. Thermal unfolding and aggregation of the bipNbs were induced by the application of a thermal ramp of 20–95°C, while measuring fluorescence ratios (F350/F330) and light scattering. Via the PR. ThermControl v2.0.4 the melting (*T*_m_) and aggregation (*T*_Agg_/*T*_turbidity_) temperature was determined.

### Viruses

All experiments with SARS‐CoV‐2 viruses were conducted in Biosafety Level 3 laboratories and approved by the Regierungspräsidium Tübingen (UNI.FRK.05.22‐101; UNI.TÜK.44.03). SARS‐CoV‐2 B.1 (SARS‐CoV‐2 Tü1 or SARS‐CoV‐2 Muc‐IMB‐1) and SARS‐CoV‐2 B.1.351 (Beta) were isolated from patient samples and variant identity was confirmed by next‐generation sequencing of the entire viral genome as described in (Ruetalo *et al*, 2021) and (Becker *et al*, 2021), respectively. SARS‐CoV‐2 B.1.617.2 (Delta) was isolated from a throat swab collected in May 2021 at the Institute for Medical Virology and Epidemiology of Viral Diseases, University Hospital Tübingen, from a PCR‐positive patient. Forty microliters of patient material was diluted in medium and used directly to inoculate 150,000 Caco‐2 cells in a six‐well plate. Seventy‐two hours post‐infection, the supernatant was collected, centrifuged, and stored at −80°C. After two consecutive passages, an RNA sample from the supernatant was prepared, and NGS confirmed that the clinical isolate belongs to the lineage B.1.617.2. Caco‐2 cell infection with and SARS‐CoV‐2 B.1.617.2 (Delta) was detected by Western blotting, using sera from a convalescent patient. Multiplicity of infection determination (MOI) was conducted by titration using serial dilutions of both virus stocks. The number of infectious virus particles per millimeter was calculated as (MOI × cell number)/(infection volume), where MOI = −ln (1 − infection rate).

### Virus neutralization assay

Caco‐2 (Human Colorectal adenocarcinoma, ATCC HTB‐37) cells were cultured at 37°C with 5% CO_2_ in DMEM containing 10% FCS, 2 mM l‐glutamine, 100 μg/ ml penicillin‐streptomycin and 1% NEAA.

Neutralization assays using clinical isolates (Fig 3A–C) were performed as described in (Becker *et al*, 2021; Ruetalo *et al*, 2021). Briefly, cells were co‐incubated with the respective clinical isolate SARS‐CoV‐2 B.1, SARS‐CoV‐2 B.1.351 (Beta), or SARS‐CoV‐2 B.1.617.2 (Delta) at MOI of ~1.0 and serial dilutions of the bipNb from 5 µM to 0.064 nM. Forty‐eight hours post‐infection, cells were fixed with 80% acetone, and immune fluorescence (IF) staining was performed using an anti‐SARS‐CoV‐2 nucleocapsid antibody (GeneTex, Cat No. GTX135357) and goat anti‐rabbit Alexa594‐conjugated secondary antibody. Cells were counterstained with DAPI solution and images were taken with the Cytation3 (BioTek). Infection rates were calculated as the ratio of DAPI‐positive over Alexa594‐positive cells, which were automatically counted by the Gen5 software (BioTek). Inhibitory concentration 50 (IC_50_) was calculated as the half‐maximal inhibitory dose using four‐parameter nonlinear regression (GraphPad Prism).

### *In vivo* infection experiments

Transgenic (K18‐hACE2)2Prlmn mice were purchased from The Jackson Laboratory and bred and kept under specific pathogen‐free conditions in the animal facilities of the University Medical Center Freiburg. Hemizygous 8–14‐week‐old animals of both sexes were used in accordance with the guidelines of the Federation for Laboratory Animal Science Associations and the National Animal Welfare Body. All experiments were in compliance with the German animal protection law and approved by the animal welfare committee of the Regierungspraesidium Freiburg (permit G‐20/91). Mice were anesthetized using isoflurane and treated intranasally (i.n.) with 20 µg of NM1251, NM1267, or NM1268 7 h prior to infection with 3 × 10^3^ PFU of the respective SARS‐CoV‐2 isolate (SARS‐CoV‐2 B.1, SARS‐CoV‐2 B.1.351 [Beta], and SARS‐CoV‐2 B.1.617.2 [Delta]) in 40 µl PBS containing 0.1% BSA. Infected mice were monitored for weight loss and clinical signs of disease for 14 days and sacrificed if severe symptoms were observed or body weight loss exceeded 25% of the initial weight. Superficial nasal swabs were taken on days 1, 2, and 3 post‐infection. Swabs were collected in OptiMEM containing 0.3% BSA and titers determined by plaque assay using Vero E6 cells. Infected ketamine/xylazine‐anesthetized mice were prepared for histological analyses by transcardial perfusion with 15 ml of 4% formaldehyde solution and stored in 4% formaldehyde at 4°C until organs were processed further. All experiments were performed under BSL3 conditions.

### Hematoxylin and eosin (H&E) staining and *in situ* hybridization (ISH)

Lung tissue was routinely embedded in paraffin and H&E staining was performed from 4‐µm‐thick lung tissue sections by using the Tissue‐Tek^®^ Prisma (Sakura). To detect SARS‐CoV‐2 RNA (plus‐strand RNA), 4‐µm‐thick lung tissue sections, including negative and positive controls, were hybridized using specific probes for SARS‐CoV‐2 (Advanced Cell Diagnostics ACD) followed by the RNAscope 2.5 HD Detection Kit Red from ACD according to the manufacturer's protocol. Quantitation of tissue damage including inflammation was defined as grade 0: no damage, grade 1: 1–10%, grade 2: 10–20%, grade 3: 20–50%, grade 4: 50–80% of lung tissue was involved.

### Analyses and statistics

Graph preparation and statistical analysis was performed using the GraphPad Prism Software (Version 9.0.0 or higher).

## Author contributions

Study design: TRW, DS, MiS, MaS, and UR; Nb biochemical characterization: TRW, PDK, BT, and DIF; Multiplex binding assay: DJ, MB, NSM; Virus neutralization assays: NR, MiS; mouse infection experiments: DS, JB, and AO; histopathological analysis and *in situ* hybridization: KK and MSa; Data analysis and statistical analysis: TRW, DS, JB, NR, MiS, KK, MaS, and UR; Manuscript drafting: TRW and UR; Study supervision: MaS, MiS, and UR; Manuscript reviewing and editing: All authors.

## Supporting information



Appendix

Expanded View Figures PDF

## Data Availability

No data that require deposition in a public database have been generated.
