# Supplementary material for: Biparatopic nanobodies protect mice from lethal challenge with SARS‐CoV‐2 variants of concern
Source: EMBO Rep. 2021 Dec 20;23(2):e53865. doi: 10.15252/embr.202153865 (PMC8811630; doi:10.15252/embr.202153865)
Supplement: Supplementary file 1 — Appendix [file EMBR-23-e53865-s003.pdf]

Appendix:

Biparatopic Nanobodies protect mice from lethal challenge with SARS-CoV-2 variants of concern

Teresa R. Wagner[1,2]*, Daniel Schnepf[3]*, Julius Beer[3]*, Natalia Ruetalo[4]*, Karin Klingel[5], Philipp D. Kaiser[2], Daniel Junker[2], Martina Sauter[5], Bjoern Traenkle[2], Desiree I. Frecot[1,2], Matthias Becker[2], Nicole Schneiderhan-Marra[2], Annette Ohnemus[3], Martin Schwemmle[3,6], Michael Schindler[4], Ulrich Rothbauer[1,2,7#]

Addresses

[1] Pharmaceutical Biotechnology, Eberhard Karls University, Tübingen, Germany

[2] NMI Natural and Medical Sciences Institute at the University of Tübingen, Reutlingen, Germany

[3] Institute of Virology, Medical Center University Freiburg, Freiburg, Germany

[4] Institute for Medical Virology and Epidemiology of Viral Diseases, University Hospital Tübingen, Tübingen, Germany

[5] Institute for Pathology and Neuropathology, University Hospital Tübingen, Tübingen, Germany

[6] Faculty of Medicine, University of Freiburg, Freiburg, Germany

[7] Cluster of Excellence iFIT (EXC2180) "Image-Guided and Functionally Instructed Tumor Therapies", Eberhard Karls University, Tübingen, Germany

* authors contributed equally to this work

# corresponding author

Correspondence:

Prof. Dr. Ulrich Rothbauer, NMI Natural and Medical Sciences Institute at the University of Tübingen

Markwiesenstr. 55, 72770 Reutlingen, Germany.
E-mail: ulrich.rothbauer@uni-tuebingen.de
Phone: +49 7121 51530-415
Fax: +49 7121 51530-816
Orcid ID: 0000-0001-5923-8986

Table of Content

Appendix Tables..4

Appendix Table S1. Nb combinations for bipNbs 4

Appendix Table S2. Primer Sequences...5

Appendix Tables

Appendix Table S1. Nb combinations for bipNbs

Biparatopic (Bip)/ bivalent Nb	Single Nb combination
NM1267	NM1230-$(G_4S)_4$-NM1226
NM1268	NM1228-$(G_4S)_4$-NM1226
NM1251 (Control)	Pep-Nb-$(G_4S)_4$-Pep-Nb

Appendix Table S2. Primer Sequences

Primer	Sequence 5’-3’
NM1228Nfor	GGACGTCTCAACTCTGACGTGCAGCTGGTGGAAT
NM1228Nrev	CACCACCGCCAGATCCACCGCCACCTGATCCTCCGCCTCCTGAGGACACGGTGACCAGGGACCC
NM1226Cfor	GGTGGATCTGGCGGTGGTGGAAGTGGTGGCGGAGGTAGTCAGGTGCAGCTGGTGGAAT
NM1226Crev	GGGGAATTCAGTGATGGTGATGGTGGTGTGAGGACACGGTGACCGGGGCC
RBDfor	ATATCTAGAGCCACCATGTTCGTGTTTCTGG
RBDrev	AAGATCTGCTAGCTCGAGTCGC
N501Yfor	GGCTTTCAGCCCACATATGGCGTGGGCTATCAGC
N501Yrev	CCACGCCATATGTGGGCTGAAAGCCGTAG
L452Rfor	GGCAACTACAATTACCGGTACCGGCTGTTCCGGAAG
L452Rrev	CGGTACCGGTAATTGTAGTTGCCGCCG
V367Ffor	CGACTACTCCTTCCTGTACAACTCCGCCAGCTTC
V367Frev	CGGAGTTGTACAGGAAGGAGTAGTCGGCCACGCA
T478Kfor	CCGGCAGCAAGCCTTGTAACGGCGTGGAAG
T478Krev	CGTTACAAGGCTTGCTGCCGGCCTGATAGA
E484Kfor	GTAACGGCGTGAAAGGCTTCAACTGCTACTTCCC
E484Krev	GCAGTTGAAGCCTTTCACGCCGTTACAAGGGGT
K417Nfor	GACAGACAGGCAACATCGCCGACTACAACTACAAGC
K417Nrev	GTTGTAGTCGGCGATGTTGCCTGTCTGTCCAGGG
K417Tfor	GACAGACAGGCACCATCGCCGACTACAACTACAAG
K417Trev	GTTGTAGTCGGCGATGGTGCCTGTCTGTCCAGGGG
