# Supplementary material for: Biparatopic nanobodies protect mice from lethal challenge with SARS‐CoV‐2 variants of concern
Source: EMBO Rep. 2021 Dec 20;23(2):e53865. doi: 10.15252/embr.202153865 (PMC8811630; doi:10.15252/embr.202153865)
Supplement: Supplementary file 2 — Expanded View Figures PDF [file EMBR-23-e53865-s002.pdf]

Expanded View Figures

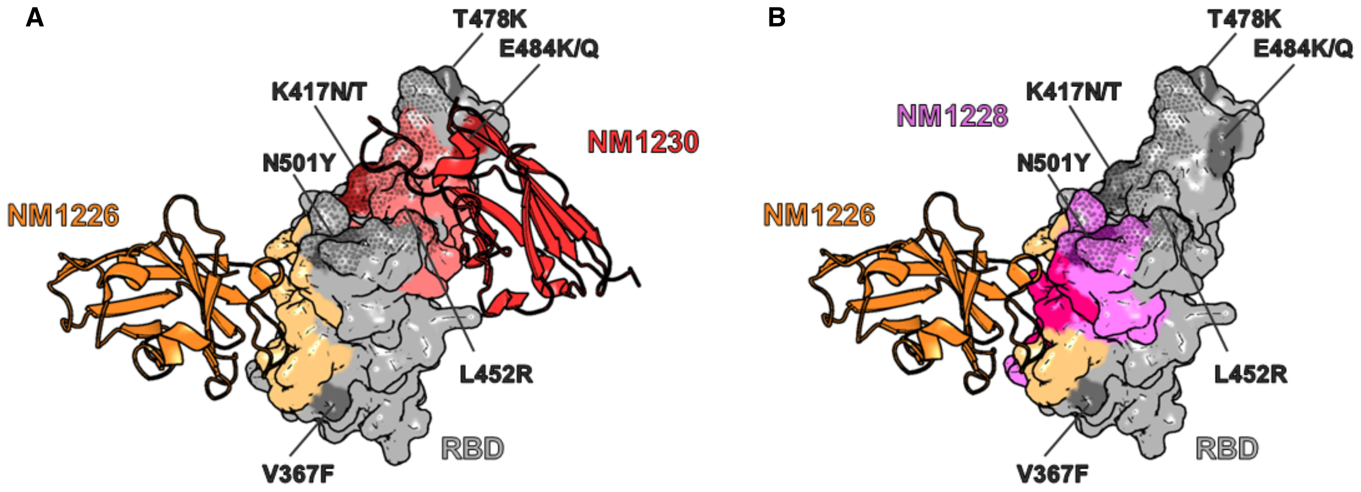

C

Pangolin (WHO)	RBD Mutation
**B.1.1.7 (Alpha)**	N501Y
**B.1.351 (Beta)**	K417N E484K N501Y
**P1 (Gamma)**	K417T E484K N501Y
**B.1.617.2 (Delta)**	L452R T478K
**B.1.429 (Epsilon)**	L452R
**P3 (Theta)**	E484K N501Y
**B.1.617.1 (Kappa)**	L452R E484Q
**A.23.1**	V367F

Figure EV1. Influence of RBD mutations on bipNb binding.

A NM1267-binding epitope on RBD derived from crystal structure of single RBD:Nb complexes NM1226 (orange, PDB 7NKT) and NM1230 (red, PDB 7B27) (Wagner *et al*, 2021). NM1267-forming single Nbs are shown as cartoon with their corresponding binding epitopes on the RBD surface in light orange and light red, respectively. In addition, the ACE2 interaction site on RBD is illustrated as dotted surface. Mutations on RBD of identified SARS-CoV-2 variants, including B.1.1.7 (Alpha), B.1.351 (Beta), P1 (Gamma), B.1.617.2 (Delta), B.1.429 (Epsilon), P3 (Theta), B.1.617.1 (Kappa), and A.23.1 are highlighted in dark gray or dark red and labeled, respectively.

B NM1268-binding epitope on RBD derived from crystal structure of the RBD:Nb complex for NM1226 (orange, PDB 7NKT) and HDX-MS analysis for NM1228 (purple) (Wagner *et al*, 2021). NM1268-forming single Nb NM1226 is shown as cartoon with its corresponding binding epitope on the RBD surface in light orange. Binding epitope of NM1228 is illustrated in purple, while shared binding sites of NM1226 and NM1228 are illustrated in pink. In addition, the ACE2 interaction site on RBD is illustrated as a dotted surface. Mutations on RBD of identified VOCs, including B.1.1.7 (Alpha), B.1.351 (Beta), P1 (Gamma), B.1.617.2 (Delta), B.1.429 (Epsilon), P3 (Theta), B.1.617.1 (Kappa), and A.23.1 are highlighted in dark gray or dark purple and labeled, respectively.

C Table summarizing mutations on RBD of different SARS-CoV-2 VOCs.

© 2021 The Authors

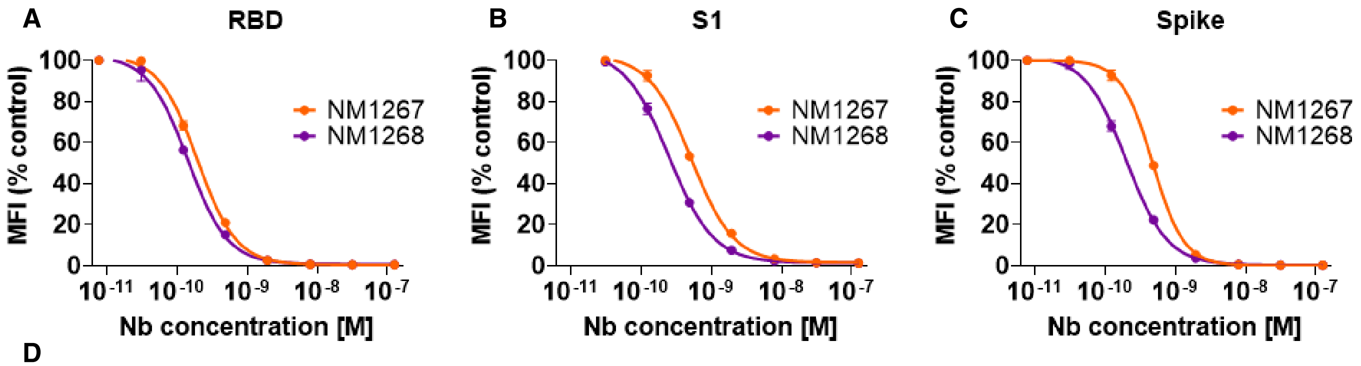

D

	IC_{50} [nM] ACE2:RBD Inhibition	IC_{50} [nM] ACE2:S1 Inhibition	IC_{50} [nM] ACE2:Spike Inhibition
**NM1267**	0.19	0.13	0.48
**NM1268**	0.14	0.06	0.20

Figure EV2. Biparatopic NM1267 and NM1268 compete with ACE2.

A–C Results from multiplex ACE2 competition assay are shown for the three spike-derived antigens: RBD (A), S1-domain (S1) (B), and homotrimeric spike (Spike) (C). Color-coded beads coated with the respective antigens were co-incubated with biotinylated ACE2 and dilution series of NM1267 and NM1268 (8 pM to 126 nM) followed by measuring residual binding of ACE2. MFI signals were normalized to the maximum detectable signal per antigen given by the ACE2-only control. IC_{50} values were calculated from a four-parametric sigmoidal model. Data are presented as mean ± SD of three technical replicates.

D Table summarizing IC_{50} values of the multiplex ACE2 competition assay obtained for NM1267 and NM1268.

Figure EV3. Intranasal application of NM1267 protects K18-hACE2 mice against B.1.351 (Beta)-induced disease, reduces mortality, and lung tissue damage.

A Schematic illustration of treatment scheme.

B Hemizygous K18-hACE2 mice were treated intranasally with 20 µg of NM1251 (*n* = 9) or NM1267 (*n* = 6) 7 h prior to infection with 3×10^3 PFU SARS-CoV-2 B.1.351 (Beta). Weight loss (left) and survival (right) were monitored for 14 days. Dashed line indicates humane end point and symbols for weight loss (left) represent mean ± SEM. ****$P < 0.0001$, by two-way ANOVA with Sidak's multiple comparison test (left) and *$P < 0.05$, by log-rank test for survival (right).

C Serial tissue sections revealed severe inflammation (H&E) and numerous widespread SARS-CoV-2 RNA-positive alveolar epithelia cells and macrophages (*in situ* hybridization [ISH]) in lungs of infected, control-treated mice. In infected and NM1267 bipNb-treated animals no inflammation or only small focal areas with inflammation and a few SARS-CoV-2 RNA-positive cells were observed. A negative control is displayed in Fig 5A and B. Scale bars represent 1 mm and 200 µm, respectively. Quantitation of lung damage was done in *n* = 3 animals per group and grading score of individual animals is presented as mean ± SD with **$P < 0.01$, by unpaired *t*-test.

© 2021 The Authors

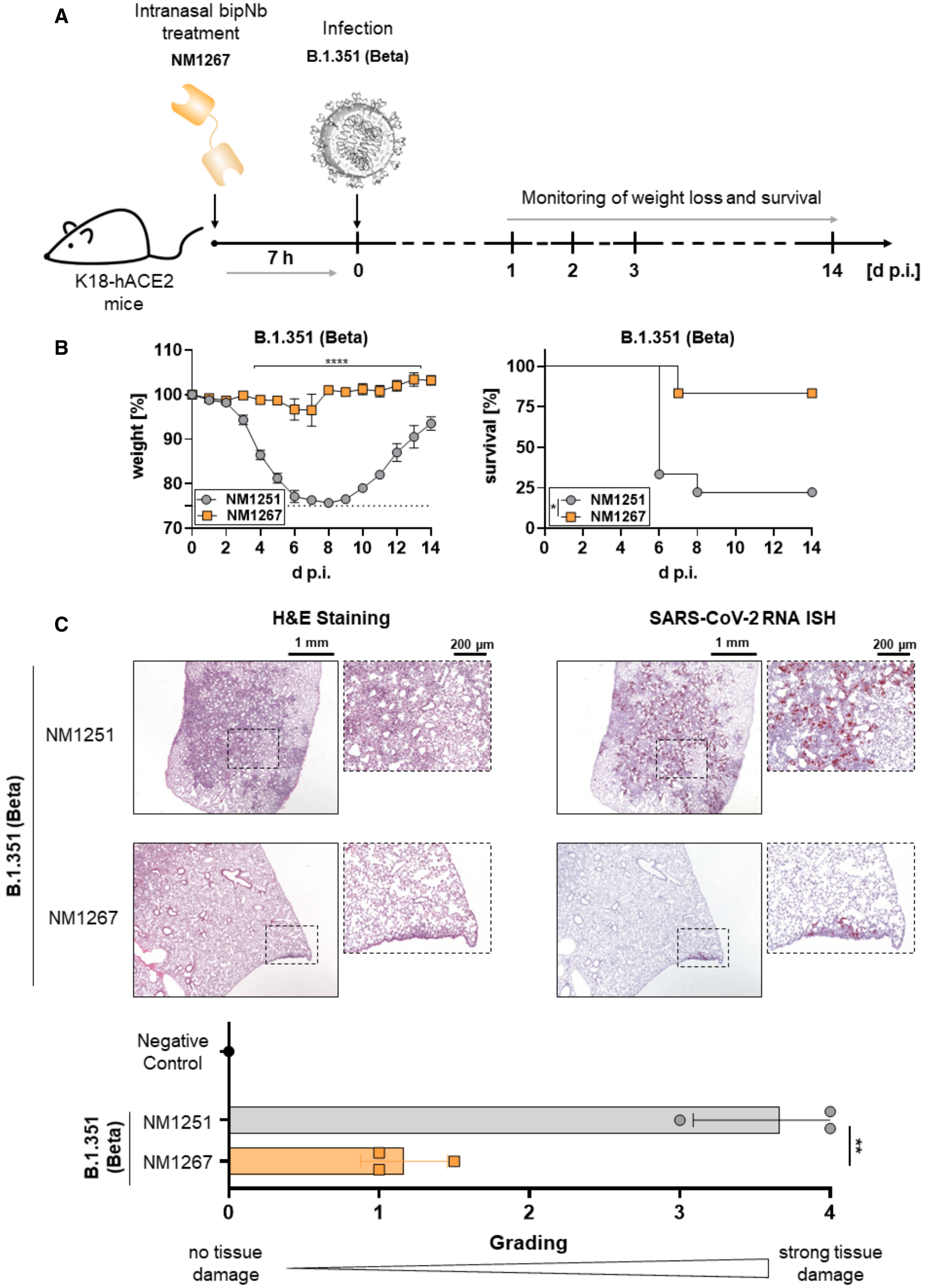
A
Intranasal bipNb treatment
NM1267
Infection
B.1.351 (Beta)
Monitoring of weight loss and survival
K18-hACE2 mice
7 h
0
1
2
3
14
[d p.i.]
B
B.1.351 (Beta)
weight [%]
****
NM1251
NM1267
d p.i.
B.1.351 (Beta)
survival [%]
*
NM1251
NM1267
d p.i.
C
H&E Staining
SARS-CoV-2 RNA ISH
1 mm
200 µm
B.1.351 (Beta)
NM1251
NM1267
Negative Control
B.1.351 (Beta)
NM1251
NM1267
**
Grading
no tissue damage
strong tissue damage

Figure EV3.
