# Supplementary material for: Biparatopic nanobodies protect mice from lethal challenge with SARS‐CoV‐2 variants of concern
Source: EMBO Rep. 2021 Dec 20;23(2):e53865. doi: 10.15252/embr.202153865 (PMC8811630; doi:10.15252/embr.202153865)
Supplement: Supplementary file 3 [file EMBR-23-e53865-s001.pdf]

EMBO reports

Biparatopic Nanobodies protect mice from lethal challenge with SARS-CoV-2 variants of concern

Teresa Wagner, Daniel Schnepf, Julius Beer, Natalia Ruetalo, karin klingel, Philipp Kaiser, Daniel Junker, Martina Sauter, Bjoern Traenkle, Desiree Frecot, Matthias Becker, Nicole Schneiderhan-Marra, Annette Ohnemus, Martin Schwemmle, Michael Schindler and Ulrich Rothbauer,
DOI: 10.15252/embr.202153865

Corresponding author(s): Ulrich Rothbauer (ulrich.rothbauer@uni-tuebingen.de)

Review Timeline:

Submission Date:	13th Oct 21
Editorial Decision:	29th Nov 21
Revision Received:	30th Nov 21
Accepted:	6th Dec 21

Editor: Martina Rembold

Transaction Report:

(Note: With the exception of the correction of typographical or spelling errors that could be a source of ambiguity, letters and reports are not edited. Depending on transfer agreements, referee reports obtained elsewhere may or may not be included in this compilation. Referee reports are anonymous unless the Referee chooses to sign their reports.)

1st Editorial Decision **29th Nov 2021**

Dear Ulrich,

Thank you for the submission of your research manuscript to our journal. I have already informed you that we have now received the full set of referee reports (copied again below).

As you will see, the referees acknowledge that the findings are potentially interesting and support publication after a relatively minor revision. We would therefore like to invite you to revise your manuscript with the understanding that the referee concerns (as detailed above and in their reports) must be fully addressed and their suggestions taken on board. Please address all referee concerns in a complete point-by-point response.

Revised manuscripts should be submitted within three months of a request for revision. In this case the revisions seem rather minor and I would like to inform you of our acceptance deadline for online publication in 2021, which is December 7th. For a publication in 2021, we would thus need to receive your revised manuscript before this date.

Since only minor revisions are required, we have already performed our quality controls on the submitted manuscript to streamline the process.

I will list below general information on the format and resubmission of the manuscript to EMBO Reports and in a second paragraph I will list more specific information items that need your attention.

A) When submitting your revised manuscript, we will require:

1) a .docx formatted version of the manuscript text (including legends for main figures, EV figures and tables). Please make sure that the changes are highlighted to be clearly visible.

2) individual production quality figure files as .eps, .tif, .jpg (one file per figure).
Please download our Figure Preparation Guidelines (figure preparation pdf) from our Author Guidelines pages https://www.embopress.org/page/journal/14693178/authorguide for more info on how to prepare your figures.

3) a .docx formatted letter INCLUDING the reviewers' reports and your detailed point-by-point responses to their comments. As part of the EMBO Press transparent editorial process, the point-by-point response is part of the Review Process File (RPF), which will be published alongside your paper.

4) a complete author checklist, which you can download from our author guidelines (<https://www.embopress.org/page/journal/14693178/authorguide>). Please insert information in the checklist that is also reflected in the manuscript. The completed author checklist will also be part of the RPF.

5) We replaced Supplementary Information with Expanded View (EV) Figures and Tables that are collapsible/expandable online. A maximum of 5 EV Figures can be typeset. EV Figures should be cited as 'Figure EV1, Figure EV2" etc... in the text and their respective legends should be included in the main text after the legends of regular figures.

- For the figures that you do NOT wish to display as Expanded View figures, they should be bundled together with their legends in a single PDF file called *Appendix*, which should start with a short Table of Content. Appendix figures should be referred to in the main text as: "Appendix Figure S1, Appendix Figure S2" etc. See detailed instructions regarding expanded view here: <https://www.embopress.org/page/journal/14693178/authorguide#expandedview>

- Additional Tables/Datasets should be labeled and referred to as Table EV1, Dataset EV1, etc. Legends have to be provided in a separate tab in case of .xls files. Alternatively, the legend can be supplied as a separate text file (README) and zipped together with the Table/Dataset file.

6) We would also encourage you to include the source data for figure panels that show essential data. Numerical data should be provided as individual .xls or .csv files (including a tab describing the data). For blots or microscopy, uncropped images should be submitted (using a zip archive if multiple images need to be supplied for one panel). Additional information on source data and instruction on how to label the files are available <https://www.embopress.org/page/journal/14693178/authorguide#sourcedata>.

7) Our journal encourages inclusion of *data citations in the reference list* to directly cite datasets that were re-used and obtained from public databases. Data citations in the article text are distinct from normal bibliographical citations and should directly link to the database records from which the data can be accessed. In the main text, data citations are formatted as follows: "Data ref: Smith et al, 2001" or "Data ref: NCBI Sequence Read Archive PRJNA342805, 2017". In the Reference list, data citations must be labeled with "[DATASET]". A data reference must provide the database name, accession number/identifiers and a resolvable link to the landing page from which the data can be accessed at the end of the reference.

Further instructions are available at <https://www.embopress.org/page/journal/14693178/authorguide#referencesformat>.

8) As part of the EMBO publication's Transparent Editorial Process, EMBO reports publishes online a Review Process File to accompany accepted manuscripts. This File will be published in conjunction with your paper and will include the referee reports, your point-by-point response and all pertinent correspondence relating to the manuscript.

You are able to opt out of this by letting the editorial office know (emboreports@embo.org). If you do opt out, the Review Process File link will point to the following statement: "No Review Process File is available with this article, as the authors have chosen not to make the review process public in this case."

B) Other, specific items:

- Please reduce the number of keywords to 5.

- Data Availability section: This section is meant to either refer to data deposited in a public repository or to state that no data that requires deposition in a public database has been generated. Please replace the current statement with one along these lines.
See also < https://www.embopress.org/page/journal/14693178/authorguide#dataavailability>).

- BioRxiv references should be clearly marked. The citation in the text is: (preprint: NAME1 et al, YEAR); in the reference list: Author NAME1, Author NAME2 (YEAR) article title. bioRxiv doi [PREPRINT].

- Figure 3D-F summarizes data from two replicates. Please show the individual datapoints instead of mean and SEM, if the data rest on two replicates.

- Funding: please add all funding sources listed in the manuscript also in the relevant fields in the online submission system.

- Please double-check the differentiation between the contributions of Martina Sauter, Martin Schwemmle, and Michael Schindler in the Author contributions section.

- Appendix: If you wish to keep the Supplementary information as Appendix instead of converting it to EV figures, please add a table of content with page numbers. Moreover, please follow the nomenclature Appendix Figure S# and Appendix Table S#.

- Appendix Fig. S3C: Please add scale bars and define their size in the legend. Please also add labels for the H&E staining and the SARS-CoV-2 RNA ISH. Do you also have images for the negative control similar to what is shown in Fig. 5?

- Our production/data editors have asked you to clarify several points in the figure legends (see attached document). Please incorporate these changes in the attached word document and return the revised file with tracked changes with your final manuscript submission. I have also taken the liberty to make some changes to the Abstract. Please review these changes and further shorten title and abstract.

- Finally, EMBO reports papers are accompanied online by A) a short (1-2 sentences) summary of the findings and their significance, B) 2-3 bullet points highlighting key results and C) a synopsis image that is 550x200-600 pixels large (width x height) in .png format. You can either show a model or key data in the synopsis image. Please note that the size is rather small and that text needs to be readable at the final size. Please send us this information along with the revised manuscript.

We would also welcome the submission of cover suggestions, or motifs to be used by our Graphics Illustrator in designing a cover.

I look forward to seeing a revised version of your manuscript when it is ready. Please let me know if you have questions or comments regarding the revision.

Kind regards,

Martina Rembold, PhD
Senior Editor
EMBO reports

****

Referee #1:

The authors report the generation and detailed characterization of a bispecific nanobody fusion construct (NM1268) which was compared with a previously described bispecific nanobody fusion construct (NM1267) that shares NM1226 as one of the building blocks. Both constructs compete with ACE2 binding in vitro, remain homogenous after 10 days accelerated aging at 37 degrees Celsius and have aggregation and melting temperature of 50-57 degrees Celsius. NM1268 binds with higher affinity than NM1267 to recombinant RBD derived from the Delta variant and neutralizes this variant at lower concentrations than NM1267, which is in line with the binding region of NM1228 and NM1230 on the RBD. Intranasal administration of NM1267, at a dose of 1mg/kg, to k18-hACE2 transgenic mice 7h before challenge with SARS-CoV2 B.1 is associated with reduced lung inflammation, reduced lung viral RNA based on in situ RNA hybridization, and increased survival. Prophylactic intranasal administration of NM1268 at 1 mg/kg provided better protection than NM1267 against morbidity and mortality of k18-hACE2 transgenic mice after challenge with a Delta variant virus.
This is a well-written manuscript with clearly presented data on the in vitro and in vivo antiviral potential of 2 biparatopic nanobody fusion constructs that target SARS-CoV-2 RBD. Demonstration of antiviral potential in vitro and in vivo against different SARS-CoV-2 VoCs is convincing. The manuscript is of interest to the broader scientific community. A therapeutic (meaning upon administration after challenge) benefit of the NBs was not demonstrated.
Remarks:
1. In the abstract it is mentioned that bipNb-treated mice secreted less infectious virus via their nostrils. This was, however, only documented in 1 experiment with bispecific NM1267. This reduction was also only observed on day 1 after infection, suggesting that NM1267 had a direct neutralizing effect on the inoculum and less so on newly produced virus in the URT of challenged mice. The statement on the nasal virus shedding in the abstract should be adapted or omitted.
2. The statement in line 219: "... solely Hanke et al. followed a similar strategy ..." suggests that the authors overlooked the work of Koenig et al (Science et al. 2021). Please adapt the statement.

Minor remarks:
1. Line 51: active against what? Please clarify.
2. Lines 84, 222, and 225: please specify which one of the 2 Hanke et al. reports is referred to here.
3. Line 410: please check the concentration range: panel A right mentions 20-2.5 nM.
4. Line 162: please add that this significant difference was observed on day 1 after infection.
5. In the discussion it is worth mentioning that humanization of the NBs would likely be needed if clinical development were to be pursued.
6. Line 333. Please specify the source of the rabbit anti-N antibody.

Referee #2:

The paper presented by Wagner et al. describes the development and pre-clinical testing of two bi-paratopic nanobodies raised against SARS-CoV-2. Broad neutralization capacity was achieved by generating two fusion constructs, both involving a nanobody addressing a highly conserved epitope that does not bear mutations in variants of concern, fused to nanobodies binding to the (more variable) ACE2 interaction site on the RBD. This approach yielded highly affine (due to avidity), small, stable and well-expressed biomolecules, which effectively neutralize all circulating variants of concern. The utility of the molecules as potential prophylactic agents was successfully demonstrated in a human ACE2 transgenic mice model.
The manuscript excels in terms of analytical rigor and data quality. It is very well written (including introduction and discussion, which embed the findings in a very rapidly evolving field), has good figures and a very clear message.
In my opinion, there is very little to be criticized about this work. Obviously, the approach of using fused nanobodies for passive immunization is not unique and had been described in other contexts in recent months. The novelty lies in the use of two different nanobodies covering non-overlapping epitopes on the ACE2 interaction part of the RBD fused to a "constant" nanobody binding the "cryptic" epitope, thereby reaching outstandingly broad neutralization capacity, when both molecules are used as a tandem.
Minor comments
Lines 127 ff: The authors occasionally found their bipNbs to bind stronger to some variants of concern than to the original (B.1) RBD. In particular, the 100 x increased affinity of NM1268 against the DELTA variant is astonishing. How can the authors explain this result? The individual nanobodies were initially raised against the original B.1 variant.
Along a similar axis: did the authors attempt to determine affinities of the biNbs against the entire spike protein? It had been observed at several instances that affinities determined against the isolated RBD can differ from those determined with the intact spike protein. The latter value is likely of higher relevance to neutralization.
Lines 217ff: The authors claim their approach of generating bipNbs as being rather unique. However, this approach has been chosen by others, who are not cited in this context (e.g. König et al, Science (2021), just to name one example). Maybe, the authors somehow want to claim that they are the first ones to perform in vivo experiments with bipNbs in mice? One may see it this way; but it is evident, that biparatopic nanobodies with similar neutralization potency in the pertinent neutralization assays (as is the case for other bipNbs that were not yet tested in animals) will very likely exhibit similar capacities in conferring a similar prophylactic effect in this mouse model.

Referee #3:

In this manuscript, the authors develop and characterize two bi-specific nanobody constructs that each target two domains on the SARS-CoV-2 Spike protein. They show that the nanobodies bind all of the Spike proteins from variants of concern with high affinity. They also show that the nanobodies neutralize infection of cells using relevant, currently circulating viral variants. Lastly, they show protective efficacy of these nanobody reagents in mouse models of severe infection. This is a thorough set of experiments to characterize these timely and innovative potential therapeutics, and to demonstrate their protective effects in a preclinical animal model. A virological study may be warranted to determine whether the bi-specific targeting strategy limits emergence of resistance mutants. Likewise, it may be valuable to determine whether the nanobodies limit cell-to-cell spread of the virus or syncytia formation. However, these future experiments are likely beyond the scope of the current study. Overall, this is a strong report that warrants rapid publication.

Point-by-point response to the Reviewers' Comments

Submission ID: EMBOR-2021-53865V2

MS TITLE: Biparatopic Nanobodies protect mice from lethal challenge with SARS-CoV-2 variants of concern

We thank all Reviewers for their detailed evaluation of our manuscript and we are pleased by the positive responses of the Reviewers mentioning "This is a well-written manuscript with clearly presented data on the in vitro and in vivo antiviral potential of 2 biparatopic nanobody fusion constructs that target SARS-CoV-2 RBD." (Reviewer 1); "The manuscript excels in terms of analytical rigor and data quality. It is very well written (including introduction and discussion, which embed the findings in a very rapidly evolving field), has good figures and a very clear message.

In my opinion, there is very little to be criticized about this work." (Reviewer 2); "This is a thorough set of experiments to characterize these timely and innovative potential therapeutics, and to demonstrate their protective effects in a preclinical animal model…. Overall, this is a strong report that warrants rapid publication." (Reviewer 3)

We are very grateful for their detailed comments, questions, and suggestions, which help us to present our results in a clearer and more comprehensive fashion. We are confident that our revised manuscript now addresses the issues raised by the Reviewers.

Referee #1

This is a well-written manuscript with clearly presented data on the in vitro and in vivo antiviral potential of 2 biparatopic nanobody fusion constructs that target SARS-CoV-2 RBD. Demonstration of antiviral potential in vitro and in vivo against different SARS-CoV-2 VoCs is convincing. The manuscript is of interest to the broader scientific community. A therapeutic (meaning upon administration after challenge) benefit of the NBs was not demonstrated.

Remarks:

1. In the abstract it is mentioned that bipNb-treated mice secreted less infectious virus via their nostrils. This was, however, only documented in 1 experiment with bispecific NM1267. This reduction was also only observed on day 1 after infection, suggesting that NM1267 had a direct neutralizing effect on the inoculum and less so on newly produced virus in the URT of challenged mice. The statement on the nasal virus shedding in the abstract should be adapted or omitted.

We agree with Referee #1 and removed this statement in the abstract of the revised manuscript.

2. The statement in line 219: "... solely Hanke et al. followed a similar strategy ..." suggests that the authors overlooked the work of Koenig et al (Science et al. 2021). Please adapt the statement.

We rephrased the text and included the proposed reference in this section of the revised manuscript.

Minor remarks:

1. Line 51: active against what? Please clarify.

For clarification, we changed this sentence in the abstract of the revised manuscript, now reading “These data suggest, that both bipNbs are broadly *active* against a variety of emerging SARS-CoV-2 VOCs and represent easily applicable drug candidates”.

2. Lines 84, 222, and 225: please specify which one of the 2 Hanke et al. reports is referred to here.

These publications can be clearly distinguished by their publication date (see below).

Hanke L, Das H, Sheward DJ, Vidakovics LP, Urgard E, Moliner-Morro A, Karl V, Pankow A, Kim C, Smith NL et al (**2021**) A bispecific monomeric nanobody induces SARS-COV-2 spike trimer dimers. bioRxiv: 2021.2003.2020.436243

Hanke L, Perez LV, Sheward DJ, Das H, Schulte T, Moliner-Morro A, Corcoran M, Achour A, Hedestam GBK, Hällberg BM (**2020**) An alpaca nanobody neutralizes SARS-CoV-2 by blocking receptor interaction. Nature communications 11: 1-9

3. Line 410: please check the concentration range: panel A right mentions 20-2.5 nM.

We thank the Referee for this remark and corrected the concentration range.

4. Line 162: please add that this significant difference was observed on day 1 after infection.

We thank the Reviewer for this notion and added the information in the text of the revised manuscript.

5. In the discussion it is worth mentioning that humanization of the NBs would likely be needed if clinical development were to be pursued.

We thank the Reviewer for this comment and included a short paragraph addressing this issue in the discussion of the revised manuscript

6. Line 333. Please specify the source of the rabbit anti-N antibody.

The missing information is now included in the revised manuscript.

Referee #2

In my opinion, there is very little to be criticized about this work. Obviously, the approach of using fused nanobodies for passive immunization is not unique and had been described in other contexts in recent months. The novelty lies in the use of two different nanobodies covering non-overlapping epitopes on the ACE2 interaction part of the RBD fused to a "constant" nanobody binding the "cryptic" epitope, thereby reaching outstandingly broad neutralization capacity, when both molecules are used as a tandem.

Minor comments

Lines 127 ff: The authors occasionally found their bipNbs to bind stronger to some variants of concern than to the original (B.1) RBD. In particular, the 100 x increased affinity of NM1268 against the DELTA variant is astonishing. How can the authors explain this result? The individual nanobodies were initially raised against the original B.1 variant.

We acknowledge that this is a very interesting point. However, since we have crystal structure data and HDX MS data of the single Nbs in complex with the B.1 RBD, we can only speculate on the impact of the characteristic mutations of the Delta VOC on Nb binding. In could be that the highly positively charged residues included in the L452R mutation alone or in combination with the T478K mutation favors binding of NM1228 by additional ionic bonds. In the light of an improved IC_{50} in the VNT and an increased survival rate in the *in vivo* experiment for NM1268, it would be indeed highly interesting to analyze the interaction of NM1226 and NM1228 with the B.1.617.2 (Delta) RBD on a structural basis to identify the molecular reason for this improved binding effect.

Along a similar axis: did the authors attempt to determine affinities of the biNbs against the entire spike protein? It had been observed at several instances that affinities determined against the

isolated RBD can differ from those determined with the intact spike protein. The latter value is likely of higher relevance to neutralization.

We agree with the Reviewer that it would be very interesting to determine the affinity of both bipNbs on the spike protein. However starting with our study, we decided to focus on the isolated RBD since this part of the spike protein could be reproducibly produced and purified for VOCs in a timely manner. We would like to emphasize that our competitive ACE2 binding assay (Figure Expanded View 2) showed that the Nbs binds equally well or even better to the S1 domain or the full length spike.

Lines 217ff: The authors claim their approach of generating bipNbs as being rather unique. However, this approach has been chosen by others, who are not cited in this context (e.g. König et al, Science (2021), just to name one example). Maybe, the authors somehow want to claim that they are the first ones to perform in vivo experiments with bipNbs in mice? One may see it this way; but it is evident, that biparatopic nanobodies with similar neutralization potency in the pertinent neutralization assays (as is the case for other bipNbs that were not yet tested in animals) will very likely exhibit similar capacities in conferring a similar prophylactic effect in this mouse model.

Indeed, we wanted to state that we used our bipNbs for *in vivo* treatment, which was previously performed only by Hanke et al. However, as the field is rapidly evolving we lower our wording accordingly and included the proposed reference in this section of the revised manuscript.

Referee #3

This is a thorough set of experiments to characterize these timely and innovative potential therapeutics, and to demonstrate their protective effects in a preclinical animal model. A virological study may be warranted to determine whether the bi-specific targeting strategy limits emergence of resistance mutants. Likewise, it may be valuable to determine whether the nanobodies limit cell-to-cell spread of the virus or syncytia formation. However, these future experiments are likely beyond the scope of the current study. Overall, this is a strong report that warrants rapid publication.

We thank the Referee for acknowledging the potential impact of our study for further developments. At this stage, we can state that we will implement the suggested experiments in further studies in order to characterize the MoA especially in the context of our bipNbs to address viral escape in more detail.

2nd Revision - Editorial Decision 6th Dec 2021

Prof. Ulrich Rothbauer
Eberhard Karls University Tuebingen
Pharmaceutical Biotechnology
Markwiesenstrasse 55
Reutlingen 72770
Germany

Dear Ulrich,

Thank you for sending the further modified manuscript text. I am now very pleased to accept your manuscript for publication in the next available issue of EMBO reports. Thank you for your contribution to our journal.

At the end of this email I include important information about how to proceed. Please ensure that you take the time to read the information and complete and return the necessary forms to allow us to publish your manuscript as quickly as possible.

As part of the EMBO publication's Transparent Editorial Process, EMBO reports publishes online a Review Process File to accompany accepted manuscripts. As you are aware, this File will be published in conjunction with your paper and will include the referee reports, your point-by-point response and all pertinent correspondence relating to the manuscript.

If you do NOT want this File to be published, please inform the editorial office within 2 days, if you have not done so already, otherwise the File will be published by default [contact: emboreports@embo.org]. If you do opt out, the Review Process File link will point to the following statement: "No Review Process File is available with this article, as the authors have chosen not to make the review process public in this case."

Should you be planning a Press Release on your article, please get in contact with emboreports@wiley.com as early as possible, in order to coordinate publication and release dates.

Thank you again for your contribution to EMBO reports and congratulations on a successful publication. Please consider us again in the future for your most exciting work.

Kind regards,
Martina

Martina Rembold, PhD
Senior Editor
EMBO reports

****

THINGS TO DO NOW:

You will receive proofs by e-mail approximately 2-3 weeks after all relevant files have been sent to our Production Office; you should return your corrections within 2 days of receiving the proofs.

Please inform us if there is likely to be any difficulty in reaching you at the above address at that time. Failure to meet our deadlines may result in a delay of publication, or publication without your corrections.

All further communications concerning your paper should quote reference number EMBOR-2021-53865V3 and be addressed to emboreports@wiley.com.

Should you be planning a Press Release on your article, please get in contact with emboreports@wiley.com as early as possible, in order to coordinate publication and release dates.

EMBO PRESS

YOU MUST COMPLETE ALL CELLS WITH A PINK BACKGROUND

PLEASE NOTE THAT THIS CHECKLIST WILL BE PUBLISHED ALONGSIDE YOUR PAPER

Corresponding Author Name: Prof. Dr. Ulrich Rothbauer
Journal Submitted to: EMBO Reports
Manuscript Number: EMBOR-2021-53865V2

Reporting Checklist For Life Sciences Articles (Rev. June 2017)

This checklist is used to ensure good reporting standards and to improve the reproducibility of published results. These guidelines are consistent with the Principles and Guidelines for Reporting Preclinical Research issued by the NIH in 2014. Please follow the journal's authorship guidelines in preparing your manuscript.

A- Figures
1. Data
The data shown in figures should satisfy the following conditions:

- the data were obtained and processed according to the field's best practice and are presented to reflect the results of the experiments in an accurate and unbiased manner.
- figure panels include only data points, measurements or observations that can be compared to each other in a scientifically meaningful way.
- graphs include clearly labeled error bars for independent experiments and sample sizes. Unless justified, error bars should not be shown for technical replicates.
- if n< 5, the individual data points from each experiment should be plotted and any statistical test employed should be justified
- Source Data should be included to report the data underlying graphs. Please follow the guidelines set out in the author ship guidelines on Data Presentation.

2. Captions
Each figure caption should contain the following information, for each panel where they are relevant:

- a specification of the experimental system investigated (eg cell line, species name).
- the assay(s) and method(s) used to carry out the reported observations and measurements
- an explicit mention of the biological and chemical entity(ies) that are being measured.
- an explicit mention of the biological and chemical entity(ies) that are altered/varied/perturbed in a controlled manner.

- the exact sample size (n) for each experimental group/condition, given as a number, not a range;
- a description of the sample collection allowing the reader to understand whether the samples represent technical or biological replicates (including how many animals, litters, cultures, etc.).
- a statement of how many times the experiment shown was independently replicated in the laboratory.
- definitions of statistical methods and measures:
  - common tests, such as t-test (please specify whether paired vs. unpaired), simple χ^2 tests, Wilcoxon and Mann-Whitney tests, can be unambiguously identified by name only, but more complex techniques should be described in the methods section;
  - are tests one-sided or two-sided?
  - are there adjustments for multiple comparisons?
  - exact statistical test results, e.g., P values = x but not P values < x;
  - definition of 'center values' as median or average;
  - definition of error bars as s.d. or s.e.m.

Any descriptions too long for the figure legend should be included in the methods section and/or with the source data.

In the pink boxes below, please ensure that the answers to the following questions are reported in the manuscript itself. Every question should be answered. If the question is not relevant to your research, please write NA (non applicable). We encourage you to include a specific subsection in the methods section for statistics, reagents, animal models and human subjects.

USEFUL LINKS FOR COMPLETING THIS FORM

http://www.antibodypedia.com
http://1degreebio.org
http://www.equator-network.org/reporting-guidelines/improving-bioscience-research-repor
http://grants.nih.gov/grants/olaw/olaw.htm
http://www.mrc.ac.uk/Ourresearch/Ethicsresearchguidance/Useofanimals/index.htm
http://ClinicalTrials.gov
http://www.consort-statement.org
http://www.consort-statement.org/checklists/view/32-consort/66-title
http://www.equator-network.org/reporting-guidelines/reporting-recommendations-for-tumo
http://datadryad.org
http://figshare.com
http://www.ncbi.nlm.nih.gov/gap
http://www.ebi.ac.uk/ega
http://biomodels.net/
http://biomodels.net/miriam/
http://jjj.biochem.sun.ac.za
https://osp.od.nih.gov/biosafety-biosecurity-and-emerging-biotechnology/
http://www.selectagents.gov/

B- Statistics and general methods

Please fill out these boxes (Do not worry if you cannot see all your text once you press return)

1.a. How was the sample size chosen to ensure adequate power to detect a pre-specified effect size?	Any details regarding sample size is given in the manuscript. All chosen sample size allows the detection of a pre-specified effect size.
1.b. For animal studies, include a statement about sample size estimate even if no statistical methods were used.	The sample size of the K18-hACE2 transgenic mice was estimated on the basis of experience with other respiratory viruses to give statistical power while minimizing animal use.
2. Describe inclusion/exclusion criteria if samples or animals were excluded from the analysis. Were the criteria pre-established?	N/A
3. Were any steps taken to minimize the effects of subjective bias when allocating animals/samples to treatment (e.g. randomization procedure)? If yes, please describe.	No
For animal studies, include a statement about randomization even if no randomization was used.	Individual mice per group (same litter) were equally distributed across different treatment groups.
4.a. Were any steps taken to minimize the effects of subjective bias during group allocation or/and when assessing results (e.g. blinding of the investigator)? If yes please describe.	Investigators were not blinded. Metric measurements were used, therefore results were not affected by subjective bias.
4.b. For animal studies, include a statement about blinding even if no blinding was done	Investigators were not blinded.
5. For every figure, are statistical tests justified as appropriate?	If a statistical test was performed, all details are given in the corresponding figure legend.
Do the data meet the assumptions of the tests (e.g., normal distribution)? Describe any methods used to assess it.	All data shown meets the requirements for the presented statistical analysis. Log-transformed values were used to calculate statistical significance for viral titers.
Is there an estimate of variation within each group of data?	Yes

Is the variance similar between the groups that are being statistically compared?	Yes

C- Reagents

6. To show that antibodies were profiled for use in the system under study (assay and species), provide a citation, catalog number and/or clone number, supplementary information or reference to an antibody validation profile. e.g., Antibodypedia (see link list at top right), 1DegreeBio (see link list at top right).	All antibody information are given in the Material and Method section.
7. Identify the source of cell lines and report if they were recently authenticated (e.g., by STR profiling) and tested for mycoplasma contamination.	Information are given in the Material and Method section.

* for all hyperlinks, please see the table at the top right of the document

D- Animal Models

8. Report species, strain, gender, age of animals and genetic modification status where applicable. Please detail housing and husbandry conditions and the source of animals.	Hemizygous 8-14 week-old B6.Cg-Tg(K18-ACE2)2Prlmn/J mice of both sexes were used. Mice were purchased from The Jackson Laboratory and bred and kept under specific-pathogen-free conditions in the animal facilities of the University Medical Center Freiburg.
9. For experiments involving live vertebrates, include a statement of compliance with ethical regulations and identify the committee(s) approving the experiments.	All experiments were in compliance with the German animal protection law and approved by the animal welfare committee of the Regierungspraesidium Freiburg (permit G-20/91).
10. We recommend consulting the ARRIVE guidelines (see link list at top right) (PLoS Biol. 8(6), e1000412, 2010) to ensure that other relevant aspects of animal studies are adequately reported. See author guidelines, under 'Reporting Guidelines'. See also: NIH (see link list at top right) and MRC (see link list at top right) recommendations. Please confirm compliance.	We confirm compliance.

E- Human Subjects

11. Identify the committee(s) approving the study protocol.	N/A
12. Include a statement confirming that informed consent was obtained from all subjects and that the experiments conformed to the principles set out in the WMA Declaration of Helsinki and the Department of Health and Human Services Belmont Report.	N/A
13. For publication of patient photos, include a statement confirming that consent to publish was obtained.	N/A
14. Report any restrictions on the availability (and/or on the use) of human data or samples.	N/A
15. Report the clinical trial registration number (at ClinicalTrials.gov or equivalent), where applicable.	N/A
16. For phase II and III randomized controlled trials, please refer to the CONSORT flow diagram (see link list at top right) and submit the CONSORT checklist (see link list at top right) with your submission. See author guidelines, under 'Reporting Guidelines'. Please confirm you have submitted this list.	N/A
17. For tumor marker prognostic studies, we recommend that you follow the REMARK reporting guidelines (see link list at top right). See author guidelines, under 'Reporting Guidelines'. Please confirm you have followed these guidelines.	N/A

F- Data Accessibility

18: Provide a "Data Availability" section at the end of the Materials & Methods, listing the accession codes for data generated in this study and deposited in a public database (e.g. RNA-Seq data: Gene Expression Omnibus GSE39462, Proteomics data: PRIDE PXD000208 etc.) Please refer to our author guidelines for 'Data Deposition'.<br><br>Data deposition in a public repository is mandatory for:<br>a. Protein, DNA and RNA sequences<br>b. Macromolecular structures<br>c. Crystallographic data for small molecules<br>d. Functional genomics data<br>e. Proteomics and molecular interactions	All information regarding data availability are available in the "Data availability" section. No data that requires dpposition in a public database has been generated.
19. Deposition is strongly recommended for any datasets that are central and integral to the study; please consider the journal's data policy. If no structured public repository exists for a given data type, we encourage the provision of datasets in the manuscript as a Supplementary Document (see author guidelines under 'Expanded View' or in unstructured repositories such as Dryad (see link list at top right) or Figshare (see link list at top right).	N/A
20. Access to human clinical and genomic datasets should be provided with as few restrictions as possible while respecting ethical obligations to the patients and relevant medical and legal issues. If practically possible and compatible with the individual consent agreement used in the study, such data should be deposited in one of the major public access-controlled repositories such as dbGAP (see link list at top right) or EGA (see link list at top right).	N/A
21. Computational models that are central and integral to a study should be shared without restrictions and provided in a machine-readable form. The relevant accession numbers or links should be provided. When possible, standardized format (SBML, CellML) should be used instead of scripts (e.g. MATLAB). Authors are strongly encouraged to follow the MIRIAM guidelines (see link list at top right) and deposit their model in a public database such as Biomodels (see link list at top right) or JWS Online (see link list at top right). If computer source code is provided with the paper, it should be deposited in a public repository or included in supplementary information.	N/A

G- Dual use research of concern

22. Could your study fall under dual use research restrictions? Please check biosecurity documents (see link list at top right) and list of select agents and toxins (APHIS/CDC) (see link list at top right). According to our biosecurity guidelines, provide a statement only if it could.	N/A
